# Supplementary material for: Protocol for the development of a core outcome set for cauda equina syndrome: systematic literature review, qualitative interviews, Delphi survey and consensus meeting
Source: BMJ Open. 2019 May 1;9(4):e024002. doi: 10.1136/bmjopen-2018-024002 (PMC6502054; doi:10.1136/bmjopen-2018-024002)
Supplement: Supplementary file 1 [file bmjopen-2018-024002supp001.pdf]

TOPIC GUIDE CES QUALITATIVE INTERVIEWS

Aims and Objectives

-To explore the patient experience of living with Cauda Equina Syndrome (CES)
-To ascertain what the patient feels are the most important outcomes that they are experiencing
-To ascertain what outcomes the patient feels are the most important to research in to improve CES management and aftercare
-To determine who should be key stakeholders
-Identify appropriate language to use for patient Delphi iterative process.

Introduction (5-10 mins)

Interviewer Name
Interviewer Occupation
Explain basic definition of CES
Explain looking for challenges experienced after the operation for CES
Explain expected intention, sensitive subjects and duration of interview and confidentiality
Confirm consent to qualitative interview

Background (<5 mins)

Interviewee name
Interviewee age
Interviewee occupation
Other medical conditions
When was your operation for CES?

Interview questions (30 mins)

How has your experience of this condition; Cauda Equina Syndrome been?
- What was it like before the back operation?
- What was it like after the back operation?

How do you feel your condition has been managed in hospital and in the community?

What were your expectations of life health-wise after the operation and what is the reality like?

Due to this condition what do you feel are the challenges to your health and wellbeing?
-bowel/bladder
-sex life
-back/ leg pain
-psychological
-anxiety/fear
-other

Would you be able to prioritise the importance of these for you now?

Was the importance of these different at earlier stages of the condition? (More relevant to those in the long term CES category)

Through this process of living with CES who else do you think has a good handle on the condition? If anyone? -Gauge other potential key stakeholders

Tell me a bit about the support you had for the condition?

Closing remarks (5 mins)

Considering your hospital, post op and follow up experience what would you have liked to change?
-support services
-more streamlined service with dedicated clinics
-research into timing for CES operations
-follow up as to the effects of long term CES

Offer the opportunity for the participant to comment on their interview transcript after transcription.
