# Supplementary material for: Protocol for the development of a core outcome set for cauda equina syndrome: systematic literature review, qualitative interviews, Delphi survey and consensus meeting
Source: BMJ Open. 2019 May 1;9(4):e024002. doi: 10.1136/bmjopen-2018-024002 (PMC6502054; doi:10.1136/bmjopen-2018-024002)
Supplement: Author's manuscript [file bmjopen-2018-024002.draft_revisions.pdf]

BMJ Open is committed to open peer review. As part of this commitment we make the peer review history of every article we publish publicly available.

When an article is published we post the peer reviewers' comments and the authors' responses online. We also post the versions of the paper that were used during peer review. These are the versions that the peer review comments apply to.

The versions of the paper that follow are the versions that were submitted during the peer review process. They are not the versions of record or the final published versions. They should not be cited or distributed as the published version of this manuscript.

BMJ Open is an open access journal and the full, final, typeset and author-corrected version of record of the manuscript is available on our site with no access controls, subscription charges or pay-per-view fees (http://bmjopen.bmj.com).

If you have any questions on BMJ Open's open peer review process please email editorial.bmjopen@bmj.com

BMJ Open

# PROTOCOL FOR THE DEVELOPMENT OF A CORE OUTCOME SET FOR CAUDA EQUINA SYNDROME: SYSTEMATIC LITERATURE REVIEW, QUALITATIVE INTERVIEWS, DELPHI SURVEY AND CONSENSUS MEETING



| | |
|---|---|
| Journal: | *BMJ Open* |
| Manuscript ID | bmjopen-2018-024002 |
| Article Type: | Protocol |
| Date Submitted by the Author: | 04-May-2018 |



| | |
|---|---|
| Complete List of Authors: | Srikandarajah, Nisaharan; University of Liverpool, Institute of Translational Medicine<br>Noble, Adam; University of Liverpool, Institute of Psychology Health and Society<br>Wilby, Martin; The Walton Centre NHS Foundation Trust, Neurosurgery<br>Clark, Simon; The Walton Centre NHS Foundation Trust, Neurosurgery<br>Williamson, Paula; University of Liverpool , Institute of Translational Medicine<br>Marson, Anthony; University of Liverpool, Institute of Translational Medicine |



| | |
|---|---|
| Keywords: | Cauda Equina Syndrome, Core Outcome Set, Delphi, Systematic Literature Review, Qualitative Interviews, Consensus |


SCHOLARONE™
Manuscripts

For peer review only

# PROTOCOL FOR THE DEVELOPMENT OF A CORE OUTCOME SET FOR CAUDA EQUINA SYNDROME: SYSTEMATIC LITERATURE REVIEW, QUALITATIVE INTERVIEWS, DELPHI SURVEY AND CONSENSUS MEETING

**Nisaharan Srikandarajah*, Adam Noble•, Martin Wilby§, Simon Clark§, Paula Williamson*, Tony Marson***

**Institute**

*Institute of Translational Medicine, University of Liverpool, Liverpool, United Kingdom; Department of Neurosurgery
§Walton Center NHS Foundation Trust, Liverpool, United Kingdom
•Institute of Psychology Health and Society, University of Liverpool, Liverpool, United Kingdom.

**Email address**

Nisaharan Srikandarajah- nishsri@liv.ac.uk

Martin Wilby- martin.wilby@thewaltoncentre.nhs.uk

Simon Clark- simon.clark@thewaltoncentre.nhs.uk

Adam Noble- adam.noble@liv.ac.uk

Paula Williamson- prw@liv.ac.uk

Tony Marson- marjon01@liv.ac.uk

**Keywords**

Cauda Equina Syndrome, Surgery, Outcomes, Core Outcome Set, Systematic Literature Review, Qualitative Interviews, Delphi Survey, Consensus Meeting

**Corresponding Author**

Mr Nisaharan Srikandarajah, Spinal Research Team, Room 2:29, Clinical Sciences Center, University of Liverpool, Lower Lane, Liverpool, UK, L9 7LJ

E-mail: nishsri@liv.ac.uk

Telephone: 01515295945

**Word Count** 4,150

For peer review only

## ABSTRACT

### Introduction

Cauda Equina Syndrome (CES) is a serious neurological condition most likely due to compression of the lumbosacral nerve roots. It can result in significant disability in young adults. The evidence is mainly from retrospective studies and there is heterogeneity in the outcomes chosen. We intend to develop a core outcome set for patients with CES. This would be the minimum set of outcomes related to all research studies in CES. It will be decided via a transparent methodology involving key stakeholders.

### Methods and Analysis

A systematic literature review and qualitative patient interviews will form a long list of outcomes. This would include all the outcomes mentioned in the literature and by the patients. The qualitative interviews will be semi structured, audio recorded, transcribed and thematically analysed with the use of NVivo version 10 to determine major themes and the outcomes described by CES patients. The next step would be to prioritise the long list of outcomes to determine which were the most important to CES patients and healthcare professionals who manage CES patients. This would be done through a two-round iterative Delphi survey and consensus meeting to decide the core outcome set for patients who have CES.

### Ethics and Dissemination

The study has ethical approval. The final core outcome set will be published and freely available through the CES patient charity websites.

### Registration Details

This study is registered with the Core Outcome Measures in Effectiveness Trials (COMET) database as study 824.


For peer review only

**Strengths and Limitations of the Study**

- Main Strength is that this study is a transparent consensus process which involves key stakeholders (patients and healthcare professionals) to decide a core outcome set for CES.
- Systematic Literature review and qualitative interviews will produce an initial list of outcomes reported by the existing studies and patients. The International Delphi and consensus meeting will prioritise these outcomes to decide the core outcome set.
- The study results rely on the assumption that stakeholders will eventually come to a consensus.
- "What" outcomes to be measured will be decided but further work is required to recommend "how" these outcomes can be measured.

## INTRODUCTION

Cauda equina syndrome (CES) is most likely due to compression of the lumbosacral nerve roots beneath the conus medullaris resulting in sensory-motor symptomatology of the lower limbs and sphincters clinically diagnosed as CES. Symptoms and signs include low back pain, unilateral or bilateral sciatica, saddle anaesthesia and motor weakness of the lower extremities with bladder and bowel dysfunction (1,2).

It is documented in the literature that timely operative decompression for CES secondary to herniated lumbar disc can lead to improved outcomes in patients (3,4,5). It is the most common emergency spine operation performed. In fact, delay or missed diagnosis of this condition incurs heavy litigation costs to the NHS at £336,000 (US $549,427) per case on average (6) as reported to the Medical Defence Union in the UK. Although a rare condition in the population mainly occurring in adults the National Spinal Task Force showed that there are roughly 1000 operations done each year for CES in the UK so it is not a rare procedure and the economic burden of severe disability is a worrying unknown for both patient quality of life and development of appropriate health services.

**Rationale for development of COS**

Through scoping searches, it was identified that there are no randomised controlled trials in

For peer review only

this condition, many retrospective observational studies and few prospective studies for the clinical outcome of patients who have CES. Most patients have had spinal surgery for CES. There is heterogeneity and inconsistency between studies in outcome reporting. The outcomes reported in the literature have not been independently validated as important to key stakeholders.

There is no defined core outcome set in CES currently and we intend to develop this core outcome set for use in CES research studies. A core outcome set defines the minimum outcomes that should be consistently measured and reported in clinical trials in a specific area of healthcare (7). With this there will be greater reporting consistency and a reduction in outcome reporting bias in healthcare studies contributing to systematic reviews and meta-analysis (8) that can lead to informed healthcare decisions.

This will be done through a systematic literature review and qualitative patient interviews to develop a long list of outcomes. These outcomes are then prioritised through two rounds of a Delphi process with key stakeholders and a consensus meeting to decide the core outcome set. This would be published and used for future research studies and improving outcome reporting in CES.

This has been done successfully in rheumatology with the OMERACT group (Outcomes Measures in Rheumatoid Arthritis Clinical Trials). This international collaboration was developed in the early 1990s involving patients in the development of core outcome sets and has improved consistency of reported trials in this speciality (8,9) http://www.omeract.org. The Core Outcome Measures in Effectiveness Trials (COMET) initiative advocates the involvement of patients and currently holds a database of on-going core outcome set developers (http://www.comet-initiative.org) to minimise duplication and foster health service user engagement (7,10).

**Scope of the COS**

We aim to identify "what" outcomes are of concern to key stakeholders using transparent methodology. We are not intending to consider how these outcomes should be measured. The 11 minimum Core Outcome Set Standards for Development (COS-STAD) recommendations are addressed in this protocol (11) (**Table 1**).

For peer review only

**Table 1.** COS-STAD recommendations

| Domain | Standard Number | Methodology | Notes |
|---|---|---|---|
| Scope Specification | 1 | The research or practice setting in which the COS is to be applied | Research studies that will inform clinical decision making |
| | 2 | The health condition(s) covered by the COS | All severities of Cauda Equina Syndrome |
| | 3 | The population(s) covered by the COS | Human adults aged 18 or above |
| | 4 | The intervention(s) covered by the COS | Decompressive spinal surgery and medical management |
| Stakeholders involved | 5 | Those who will use the COS in research | Clinical trialists in CES are healthcare professionals who manage CES patients. They are included in standard 6. |
| | 6 | Healthcare professionals with experience of patients with the condition | This will include clinicians, experts and healthcare professionals involved in CES management |
| | 7 | Patients with the condition or their representatives | Patients who have had an operation for CES will be included [12] |
| Consensus Process | 8 | The initial list of outcomes considered both healthcare professionals and patients views | Systematic Literature review [13] considered healthcare professional views. Qualitative interviews considered patient views. |
| | 9 | A scoring process and consensus definition were described a priori | Described in “Scoring” and “Analysis” section of this protocol |
| | 10 | Criteria for including/dropping/adding outcomes were described a priori | Described in “Analysis” section of this protocol |
| | 11 | Care was taken to avoid ambiguity of language used in the list of outcomes | Plain language and clinical explanations available. These will be pilot tested with patients and healthcare professionals. |

For peer review only

**Registration**

The study is registered on the COMET database as study 824 (http://www.comet-initiative.org/studies/details/824?result=true).

**METHODS AND ANALYSIS**

Development of the core outcome set will be developed in the following phases:

Phase 1: Systematic Literature Review

Phase 2: Qualitative Interviews

Phase 3: Delphi Survey

Phase 4: Consensus Meeting

***Phase 1: Systematic Literature Review***

**Research Question**

What outcomes are reported in the medical literature after surgery for CES?

**Method**

The aim of the systematic literature review is to summarise the reporting standards of clinical outcomes following surgery in CES patients using the PRISMA guidelines (14). Most patients who have CES will undergo an operation. It summarised a list of outcomes that had been mentioned in the literature and categorised them into a known taxonomy (15). Full details including search strategy, study selection criteria and results of the systematic literature review have been published (13).

***Phase 2: Qualitative Interviews***

**Research Question**

What outcomes have patients experienced after having surgery for CES and how do they feel about the management before and after surgery?

**Method**

The objectives of the qualitative interviews with CES patients are:

For peer review only

- To explore the patient experience of living with CES
- To ascertain what the patient feels are the most important outcomes that they are experiencing
- To ascertain what outcomes the patient feels are the most important to research in to improve CES management and aftercare
- To determine who should be key stakeholders
- Identify appropriate language to use for patient Delphi iterative process (16).

These interviews will be documented with audio recorded transcripts. The list of all potential outcomes from the systematic review and qualitative interviews will be placed into outcome domains by the research team to avoid repetition by qualitative method of content analysis (17). The qualitative interviews will be piloted with 2 CES patients to establish if the interview structure and technique is clear, understandable, and capable of answering the research questions. This would recognise any corrections that need to be made to interview structure or technique. Inclusion and exclusion criteria are shown in **Table 2**.

**Table 2**. Inclusion and Exclusion criteria for qualitative interviews

| **INCLUSION CRITERIA** | **EXCLUSION CRITERIA** |
|---|---|
| Adult patients | Adults unable to consent for research |
| Diagnosis of Cauda Equina Syndrome | |
| Patient underwent surgical procedure | |
| Less than 10 years since surgical procedure | |
| Ability to converse in English and to consent for research | |

**Participant Selection**

Adult patients will be selected from those coded as having a diagnosis of cauda equina syndrome in the medical records. There is an existing database of cauda equina patients who have been operated on and followed up by consultants, registrars or nurse specialists. There is no discrimination leading to a patient going to one clinic or the other; it is done by

For peer review only

next available clinic. Adult patients would be 18 or older. They will have undergone spinal surgery to remove the compressive lesion at a single tertiary NHS institution over the past 10 years. This will capture short term and long term outcomes that are deemed important to them. Time to recording outcome will be taken since initial operation for CES.

Stratified purposive sampling (18) was chosen in which the aim is to select groups that display variation in some particular phenomena but each of which is fairy homogenous so the subgroups can be compared. Characteristics known to have an impact on the outcomes being investigated have been identified through scoping searches- severity of CES (CESI and CESR) (19) then there is a subgroup about which little is known and whose circumstances and views need to be explored; short (<2 years) or long term (>2 years and <10 years) since the operation (see **Table 3**). This will produce 4 subcategories to populate. This is to prevent potential bias you may get from having many patients who presented with a severe clinical picture and poor outcomes being more forthcoming and vocal. All subcategories for the sampling frame were deemed a priority and “nesting” of male and female was done within the subcategory. Half would ideally be male and half would be female.

**Table 3.** Sampling frame with suggested quotas

| | **CESI (Cauda Equina Syndrome Incomplete)** | **CESR (Cauda Equina Syndrome with retention)** |
|---|---|---|
| **Short term <2 yrs since operation** | 8-10 participants | 8-10 participants |
| **Long term >2 yrs <10 yrs since operation** | 10-12 participants | 10-12 participants |

We already have an existing database of 200 patients, which will be updated up to current date with contact details and clinical details of presentation and management. This should produce 50 patients per category. Due to reasons such as patients who have died, long travel distance from institution, not interested in participating it is anticipated that up to 10 patients may reply from each category, which would produce up to 40 patients in total. Options will be given to be interviewed at home, via electronic media (Skype), over the phone or to attend the hospital in person. All patients who consented will be interviewed


For peer review only - http://bmjopen.bmj.com/site/about/guidelines.xhtml

until “data saturation” is reached and the research team will decide this collectively. Data saturation is the point where increasing the sample size no longer contributes to new evidence (20) and even large qualitative studies do not interview more than 50 people (21).

Sticking rigidly to a sample frame could be counter-intuitive as one patient can be data rich during the interview as opposed to interviewing 5 patients where data is not rich. The aim is to collect rich data to allow in depth analysis (20). So, although the sampling frame may serve as a guide we will not use it to start restricting participants especially at the initial stages of doing the qualitative interviews until data saturation is achieved.

An information leaflet would be sent to participants with a consent form. I would give them 3 weeks to “opt-out” of the study by returning a response slip. After this the participants will receive a phone call to confirm interest for partaking in the study, to answer any further questions and to arrange a time and location for the interview.

**Interview Format and Analysis**

A semi-structured interview format will be utilised as per our topic guide (**Supplementary file 1).** Informed consent will be obtained prior to interview where anonymity and confidentiality will be expressed. Open-ended non-leading questions on their diagnosis, management post operatively and in the community will be asked allowing the participant to recount their experiences without unnecessary interruption (21). Discussion will be directed towards outcomes of importance to the patient as seen in the topic guide. Interviewer will not divulge personal information about themselves and if any of these questions are asked they can be answered at the end of the interview session. Reflexivity is an important concept during qualitative research for striving towards objectivity and neutrality (20) and analysis of the interviews will consider if bias from the interviewer’s own beliefs may have crept in. It is anticipated that the interview will last for 45mins to an hour at each sitting to prevent participant feeling pressurised. The same interviewer (NS) would be used throughout. All interviewees will be made aware that the interviewer is a doctor not involved in their on-going care. A sample of these transcripts will be reviewed by a supervisor not involved in the qualitative interviews to confirm that they had been undertaken in a satisfactory manner. Initially transcripts will be reviewed to start identifying which outcomes are important to the patients by labelling and tagging the data. We will use



For peer review only - http://bmjopen.bmj.com/site/about/guidelines.xhtml

descriptive analysis to detect, categorize, and classify the transcripts using NVivo qualitative data analysis software version 10. Thematic charting will allow summarisation of the key outcomes of each individual transcript and overall themes whilst retaining the context and language in which it was expressed (20). The qualitative interviews will be reported as outlined by the consolidated criteria for reporting qualitative research (COREQ); a 32 item checklist (22).

***Phase 3: The Delphi Survey***

The outcomes from the systematic literature review and qualitative interviews will create a long list. This will be condensed by grouping similar outcomes into domains and conforming with the taxonomy used in the systematic literature review ([13 15]). This will be reviewed and agreed by the study team and pilot tested with the key stakeholders before the Delphi survey is distributed.

**Research Question**

Which outcomes do patients and healthcare professionals think should be included in a core outcome set for research studies on CES patients?

**Method**

In our "modified" Delphi, questioning will take place in two rounds. The condensed list of outcomes derived from the long list will be presented in the first round of the Delphi. Patients can also suggest outcomes that have not been mentioned in the first round but these will not be scored. They will be considered for inclusion into the second round of the Delphi if, as judged by the CES study team, the outcome does not reflect or is not similar to another outcome already listed. After the first round, an anonymous summary of the responses is fed back to the group. Individual participants can decide to keep their original answers or to change their opinion in the next round. This will lead to the group converging on a consensus opinion over the course of these two rounds (23). The Delphi will be done by healthcare professionals, and patients.

For peer review only - http://bmjopen.bmj.com/site/about/guidelines.xhtml

The level of anonymity will be "fully anonymised" (23) so participants do not know the identities of other individuals in the group and they will not know specific answers other individuals had given.

**Inclusion criteria**

Participants will be recruited from two key stakeholder groups: patients and healthcare professionals. All participants should be adults over 18 years of age and able to complete an online survey in the English language.

*Patients*- Participants who have had an operation for CES.

*Healthcare Professionals*- All members of the clinical team involved in directly caring for a patient with CES after surgery such as:

- Spinal surgeons
- Spinal specialist nurses
- Neuro-rehabilitation doctors

**Sampling and Recruitment**

*Patients*- At the main site the clinical care team have a pre-existing database of CES patients they have clinically managed. The clinical care team will send an invitation letter to the home address of these patients. There will be no follow up calls or further correspondence. It is the patient's decision if they wish to be involved and the invitation will contain details of the website address patients can access if they wish to find out more details regarding the study. Online patient groups for CES will be contacted internationally. A named contact for each group will act as the liaison member to circulate the participant invitation email and poster. This may include the patient groups sharing the recruitment details on social media.

*Healthcare Professionals*- The main study site has spinal MDT (multi-disciplinary team) meetings held weekly. The co-ordinator has a pre-set mailing list that goes to healthcare professionals involved in the meeting. This will be used to send the participant invitation email. The membership of national and international associations will be contacted and invited to participate. A few are listed here below as an example:

- Society of British Neurological Surgeons (SBNS)

For peer review only

- British Association of Spine Surgeons (BASS)
- Australian Spine Society
- North American Spine Association (NASS)

Known contacts of the CES study group will be contacted and invited to participate. Snowballing sampling will be used to increase the sample size. The participant invitation email/ letter will be the first contact for healthcare professionals and patients, which is a short introduction and summary of the study. If they are interested further the participant can proceed to the registration website for further details and obtain a copy of the participant information leaflet.

**Sample Size**

There are no strict recommendations for the number of participants required in a Delphi study to gain consensus (23). In general, having more participants will increase the reliability of the group judgement (24). We intend to take a pragmatic approach to sample size and would like to invite all individuals who meet the inclusion criteria as identified above. Documentation of the number invited and the number from each stakeholder group will be recorded. No further participants will be invited after the first round of the Delphi.

**Consent**

Consent will be implicit by the participant registering to take part in the Delphi process via the website.

**Questionnaires**

The questionnaire is constructed and delivered in an online format using the DelphiManager software developed by the COMET initiative. Before starting the questionnaire, the participant will be asked to clarify which of the two stakeholder groups they belong to. For each stakeholder group, specific information will be collected:

- *Patients*- Age, gender, location, surgery for CES, years since surgery for CES, employment status
- *Healthcare professionals*- Practicing Field (spinal surgeon, specialist nurse, neuro-rehabilitation etc), years in practice, location, gender

For peer review only

Following confirmation of their eligibility to participate in the study, participants will be sent an on-line link to access the first round of the Delphi process. Instructions of how to complete the questionnaire will be included at the beginning of each round. Only participants who respond to the 1st round of the Delphi will be invited to participate in 2nd round taking the assumption that if they had not participated in the first round they would be unwilling to participate in the second round. Data will be collected over at least a 4-week period for each round of the Delphi process. Participants who have not completed the survey will be sent reminders via email when they have 2 weeks, 1 week and 48 hours remaining for completion of the survey. Participants who have not completed the questionnaire within 4 weeks of the start will be deemed not to have completed that round of the Delphi. The language used by patients in the qualitative interviews will be used to help term the outcomes for the Delphi. Plain language summaries by the COMET Patient Participation, Involvement and Engagement (PoPPIE) group was used to develop the Delphi information sheet. The Delphi will be piloted with 2 participants from each stakeholder group to highlight any issues with understanding or validity.

**Scoring**

For an outcome to be included in the core outcome set there must be a majority agreement of the critical importance of the outcome and minority agreement that the outcome is not important. Therefore, in respect for an outcome to be excluded there must be majority agreement that it is not important and minority agreement that it is important (25). This is in par with the GRADE (Grading of Recommendations Assessment, Development and Evaluation) working group recommendations (http://www.gradeworkinggroup.org) (26,27). At the beginning of the Delphi, participants will be reminded the importance of completing the entire Delphi process. Round one of the Delphi study we will ask participants to rate each outcome using a 9 point Likert scale. This scoring system was chosen after previous studies and expert databases showed it differentiates the most between questionnaire items (23) (http://www.comet-initiative.org). 7-9 indicates critical importance. 4 to 6 represents outcomes that are important but not critical whilst 1 to 3 are deemed to be of limited importance. All outcomes will be carried through to second round with first round scores displayed for each outcome. Round two will present the anonymised feedback from


For peer review only - http://bmjopen.bmj.com/site/about/guidelines.xhtml

each participant stakeholder groups (patient and healthcare professionals). The feedback will show the cumulated scores from each stakeholder group for each outcome and the participant will be asked to rate the outcomes again using the same 9 point Likert scale. If they change their score on the second round they will have the opportunity to explain their reasoning for this. Outcomes which have been suggested in round 1 by the participants and deemed appropriate by the study group will then be entered in for rating in the second round by key stakeholders. After the final Delphi round, there will be a list developed from all stakeholder groups, which will be submitted to a face to face consensus meeting of key stakeholders to discuss what outcomes that should be finally included in the core outcome set. All stakeholder groups who had completed the Delphi survey will be invited to participate in the consensus meeting. Ideally, a trained facilitator would chair this meeting.

**Analysis**

Consensus that an outcome should not be included in the COS could, for example, be defined as 70% or more scoring it as 1 to 3 and fewer than 15% scoring it as 7 to 9, which is has been seen to be successful with the development of other core outcome sets (28,29) (**Table 4**). This will be done for each stakeholder group. Results at the multiple rounds of the Delphi process and consensus meeting will be documented to include number of participants invited, number completing the section, measure of each group response to an outcome leading to a comprehensive list of all outcomes that should be included in the COS CES.

**Table 4**. Definitions of a consensus

| Classification of consensus | Description | Definition |
|---|---|---|
| **IN** | Consensus that outcome should be included in the core outcome set | 70% or more participants scoring as 7 to 9 AND <15% participants scoring as 1 to 3 |
| **OUT** | Consensus that outcome should not be included in the core outcome set | 70% or more participants scoring as 1 to 3 AND <15% of participants scoring as 7 to 9 |


For peer review only - http://bmjopen.bmj.com/site/about/guidelines.xhtml

| | | |
|---|---|---|
| **NO CONSENSUS** | Uncertainty about importance of outcome | Anything else |

**Attrition**

It is expected that some participants will drop out after each round of the Delphi. Each participant will be given a unique participant number when they complete the first round of the Delphi, which will allow identification of the attrition rates between rounds. This will allow identification of participants who have completed all rounds and see if there is any difference bias between those participants who complete the process. We would compare the mean round 1 scores for the participants who completed round 1 and round 2 compared with those that dropped out after round 1.

***Phase 4: Consensus Meeting***

All participants registering for the Delphi survey will be asked whether they would be happy to attend a face to face consensus meeting involving patients and healthcare professionals. This would be set up as a tick box on the registration page for the online Delphi. A minority of participants at the consensus meeting will be invited before the Delphi survey to attend the consensus meeting but on the premise, that both rounds of the Delphi are completed. This is to make sure there is representation from certain organisations closely involved with CES patients, research or management. Most participants at the consensus meeting will be those who have completed all rounds of the Delphi and ticked their interest to attend the consensus meeting during registration.

In the development of a breast reconstruction core outcome set patients and professionals were recruited in a 2:1 ratio so that patients' views were represented preferentially as the procedure is a patient selected optional intervention (30). In our study, surgery for CES is usually done as an emergency operation in most cases and strongly recommended to patients so a 1:1 ratio would be expected but we will be pragmatic depending on our response rate. This is to maximise the number of participants involved to help achieve consensus. If there is an overwhelming response with more than 40 participants interested in attending the consensus meeting the study team will consider applying stratified

For peer review only

purposive sampling. On the day of the consensus meeting consent will be obtained from the patient participants. Results of the Delphi process will be discussed at the consensus meeting. Outcomes categorised as consensus “in” across both stakeholder groups will be included in the final core outcome set. Outcomes categorised as consensus “out” across both stakeholder groups will be excluded from the final core outcome set. Participants at the meeting can vote to accept this or to suggest outcomes from the group that may need further discussion. These outcomes plus “no consensus” outcomes will be discussed with further rounds of voting to agree the final core outcome set. If there is no agreed final core outcome set at the end of the first meeting subsequent meetings will be arranged for this to happen.

**ETHICS AND DISSEMINATION**

REC and HRA approval was obtained on the 6 December 2016 for the qualitative interviews from South Central - Hampshire A Research Ethics Committee. REC reference 16/SC/0587. REC and HRA approval was obtained on 26 March 2018 for the Delphi process and consensus meeting from North West- Greater Manchester Central Research Ethics Committee. REC reference was 18/NW/0022. We intend to publish the results of the core outcome set for CES in an open access journal. It will also be made available through the CES patient websites. Results will be disseminated through International and national presentations. The next step would be to identify the appropriate measurement instrument for each of the outcomes in the core outcome set (31). Core outcome sets are developed in a number of clinical areas and their use is advocated in the UK by the National Institute for Health Research (NIHR) Health Technology Assessment (HTA), Cochrane Reviews of the effects of Healthcare intervention (32) and by World Health Organisation (WHO) handbook for guideline development (10). The NIHR HTA has added this statement to their application form, “Where established core outcomes exist they should be included among the list of outcomes unless there is a good reason to do otherwise.” By developing the CES COS we intend to reduce outcome reporting bias, heterogeneity, and improve the quality of research studies in CES. This will allow us to synthesise the data and make more robust evidence based decisions regarding CES management.

For peer review only - http://bmjopen.bmj.com/site/about/guidelines.xhtml

**Supplementary file 1**- Topic guide

**Author Contributions**

NS, MW, SC conceived the project. TM is the principal investigator for the study. NS is the clinical research fellow responsible for management of the project, wrote the protocol and manuscript. TM, PW, AN, MW, SC provide supervision, have input in all aspects of the project and commented on drafts of the manuscript. All authors have read and approved the manuscript.

**Funding**

The paper is independent research funded by the Walton Centre Research Funds and Royal College of Surgeons Research Fellowship.

**Competing Interests**

None Declared

**REFERENCES**

1. Kostuik JP. Controversies in cauda equina syndrome and lumbar disk herniation. *Current Opinion in Orthopaedics* 1993;4(2):125-28.
2. Gardner A, Gardner E, Morley T. Cauda equina syndrome: A review of the current clinical and medico-legal position. *Eur Spine J* 2011;20(5):690-97. doi: http://dx.doi.org/10.1007/s00586-010-1668-3
3. Ahn UM, Ahn NU, Buchowski JM, et al. Cauda equina syndrome secondary to lumbar disc herniation. *Spine* 2000;25(12):1515-22. doi: http://dx.doi.org/10.1097/00007632-200006150-00010
4. Todd NV. Cauda equina syndrome: The timing of surgery probably does influence outcome. *Br J Neurosurg* 2005;19(4):301-06. doi: http://dx.doi.org/10.1080/02688690500305324
5. Srikandarajah N, Boissaud-Cooke MA, Clark S, et al. Does early surgical decompression in cauda equina syndrome improve bladder outcome? *Spine (03622436)* 2015;40(8):580-83. doi: 10.1097/BRS.0000000000000813
6. Daniels EW, Gordon Z, French K, et al. Review of medicolegal cases for cauda equina syndrome: what factors lead to an adverse outcome for the provider? *Orthopedics* 2012;35(3):200-00. doi: 10.3928/01477447-20120222-15
7. Williamson PR, Altman DG, Blazeby JM, et al. Developing core outcome sets for clinical trials: issues to consider. *Trials* 2012;13(1):132.

For peer review only

8. Kirkham JJ, Gargon E, Clarke M, et al. Can a core outcome set improve the quality of systematic reviews?–a survey of the Co-ordinating Editors of Cochrane Review Groups. *Trials* 2013;14(1):21.
9. Tugwell P, Boers M, Brooks P, et al. OMERACT: an international initiative to improve outcome measurement in rheumatology. *Trials* 2007;8(1):38.
10. Williamson PR, Altman DG, Blazeby JM, et al. The COMET (core outcome measures in effectiveness trials) initiative. *Trials* 2011;12(Suppl 1):A70.
11. Kirkham JJ, Davis K, Altman DG, et al. Core Outcome Set-STAndards for Development: The COS-STAD recommendations. *PLoS medicine* 2017;14(11):e1002447.
12. Young B, Bagley H. Including patients in core outcome set development: issues to consider based on three workshops with around 100 international delegates. *Research Involvement and Engagement* 2016;2(1):25.
13. Srikandarajah N, Wilby M, Clark S, et al. Outcomes reported after surgery for Cauda Equina Syndrome: A Systematic Literature Review. *Spine* 2018
14. Moher D, Liberati A, Tetzlaff J, et al. Preferred reporting items for systematic reviews and meta-analyses: the PRISMA statement. *PLoS medicine* 2009;6(7):e1000097.
15. Dodd S, Clarke M, Becker L, et al. A taxonomy has been developed for outcomes in medical research to help improve knowledge discovery. *Journal of clinical epidemiology* 2018;96:84-92.
16. Keeley T, Khan H, Pinfold V, et al. Core outcome sets for use in effectiveness trials involving people with bipolar and schizophrenia in a community-based setting (PARTNERS2): study protocol for the development of two core outcome sets. *Trials* 2015;16(1):47.
17. Elo S, Kyngäs H. The qualitative content analysis process. *Journal of advanced nursing* 2008;62(1):107-15.
18. Quinn Patton M. Qualitative Research John Wiley & Sons: Ltd, 2005.
19. Gleave JRW, Macfarlane R. Cauda equina syndrome: What is the relationship between timing of surgery and outcome? *Br J Neurosurg* 2002;16(4):325-28. doi: http://dx.doi.org/10.1080/02688690210000032887
20. Ritchie J, Lewis J, Nicholls CM, et al. Qualitative research practice: A guide for social science students and researchers: Sage 2013.
21. Britten N. Qualitative research: qualitative interviews in medical research. *Bmj* 1995;311(6999):251-53.
22. Tong A, Sainsbury P, Craig J. Consolidated criteria for reporting qualitative research (COREQ): a 32-item checklist for interviews and focus groups. *International journal for quality in health care* 2007;19(6):349-57.
23. Sinha IP, Smyth RL, Williamson PR. Using the Delphi technique to determine which outcomes to measure in clinical trials: recommendations for the future based on a systematic review of existing studies. *PLoS medicine* 2011;8(1):e1000393.
24. Murphy M. Consensus development methods and their use in clinical guideline development. *Health Technol Assess* 1998;2:1-88.
25. Jaeschke R, Guyatt GH, Dellinger P, et al. Use of GRADE grid to reach decisions on clinical practice guidelines when consensus is elusive. *BMJ: British Medical Journal (Online)* 2008;337
26. Atkins D, Best D, Briss PA, et al. Grading quality of evidence and strength of recommendations. *BMJ (Clinical research ed)* 2004;328(7454):1490-90.
27. Guyatt GH, Oxman AD, Vist GE, et al. GRADE: an emerging consensus on rating quality of evidence and strength of recommendations. *BMJ (Clinical research ed)* 2008;336(7650):924-26.

For peer review only

28. Harman NL, Bruce IA, Callery P, et al. MOMENT–Management of Otitis Media with Effusion in Cleft Palate: protocol for a systematic review of the literature and identification of a core outcome set using a Delphi survey. *Trials* 2013;14(1):70.
29. Haywood K, Griffin X, Achten J, et al. Developing a core outcome set for hip fracture trials. *Bone Joint J* 2014;96(8):1016-23.
30. Potter S, Holcombe C, Ward J, et al. Development of a core outcome set for research and audit studies in reconstructive breast surgery. *British Journal of Surgery* 2015;102(11):1360-71.
31. Prinsen CA, Vohra S, Rose MR, et al. How to select outcome measurement instruments for outcomes included in a "Core Outcome Set"–a practical guideline. *Trials* 2016;17(1):449.
32. Rosenbaum SE, Glenton C, Oxman AD. Summary-of-findings tables in Cochrane reviews improved understanding and rapid retrieval of key information. *Journal of clinical epidemiology* 2010;63(6):620-26.

For peer review only

IRAS: 201946 Version: 1.0 Date: 23/09/16

# TOPIC GUIDE CES QUALITATIVE INTERVIEWS

## Aims and Objectives

-To explore the patient experience of living with Cauda Equina Syndrome (CES)
-To ascertain what the patient feels are the most important outcomes that they are experiencing
-To ascertain what outcomes the patient feels are the most important to research in to improve CES management and aftercare
-To determine who should be key stakeholders
-Identify appropriate language to use for patient Delphi iterative process.

## Introduction (5-10 mins)

Interviewer Name
Interviewer Occupation
Explain basic definition of CES
Explain looking for challenges experienced after the operation for CES
Explain expected intention, sensitive subjects and duration of interview and confidentiality
Confirm consent to qualitative interview

## Background (<5 mins)

Interviewee name
Interviewee age
Interviewee occupation
Other medical conditions
When was your operation for CES?

## Interview questions (30 mins)

How has your experience of this condition; Cauda Equina Syndrome been?
- What was it like before the back operation?
- What was it like after the back operation?

How do you feel your condition has been managed in hospital and in the community?

What were your expectations of life health-wise after the operation and what is the reality like?

For peer review only

IRAS: 201946 Version: 1.0 Date: 23/09/16

Due to this condition what do you feel are the challenges to your health and wellbeing?
-bowel/bladder
-sex life
-back/ leg pain
-psychological
-**anxiety/fear**
-other

Would you be able to prioritise the importance of these for you now?

Was the importance of these different at earlier stages of the condition? (More relevant to those in the long term CES category)

Through this process of living with CES who else do you think has a good handle on the condition? If anyone? -Gauge other potential key stakeholders

Tell me a bit about the support you had for the condition?

**Closing remarks (5 mins)**

Considering your hospital, post op and follow up experience what would you have liked to change?
-support services
-more streamlined service with dedicated clinics
-research into timing for CES operations
-follow up as to the effects of long term CES

Offer the opportunity for the participant to comment on their interview transcript after transcription.

For peer review only

# PROTOCOL FOR THE DEVELOPMENT OF A CORE OUTCOME SET FOR CAUDA EQUINA SYNDROME: SYSTEMATIC LITERATURE REVIEW, QUALITATIVE INTERVIEWS, DELPHI SURVEY AND CONSENSUS MEETING



| | |
|---|---|
| Journal: | *BMJ Open* |
| Manuscript ID | bmjopen-2018-024002.R1 |
| Article Type: | Protocol |
| Date Submitted by the Author: | 25-Oct-2018 |
| Complete List of Authors: | Srikandarajah, Nisaharan; University of Liverpool, Institute of Translational Medicine<br>Noble, Adam; University of Liverpool, Institute of Psychology Health and Society<br>Wilby, Martin; The Walton Centre NHS Foundation Trust, Neurosurgery<br>Clark, Simon; The Walton Centre NHS Foundation Trust, Neurosurgery<br>Williamson, Paula; University of Liverpool , Institute of Translational Medicine<br>Marson, Anthony; University of Liverpool, Institute of Translational Medicine |
| <b>Primary Subject Heading</b>: | Surgery |
| Secondary Subject Heading: | Medical management, Patient-centred medicine, Qualitative research, Research methods, Neurology |
| Keywords: | Cauda Equina Syndrome, Core Outcome Set, Delphi, Systematic Literature Review, Qualitative Interviews, Consensus |
| | |



SCHOLARONE™
Manuscripts

# PROTOCOL FOR THE DEVELOPMENT OF A CORE OUTCOME SET FOR CAUDA EQUINA SYNDROME: SYSTEMATIC LITERATURE REVIEW, QUALITATIVE INTERVIEWS, DELPHI SURVEY AND CONSENSUS MEETING

**Nisaharan Srikandarajah*, Adam Noble•, Martin Wilby§, Simon Clark§, Paula Williamson*, Tony Marson***

**Institute**

*Institute of Translational Medicine, University of Liverpool, Liverpool, United Kingdom; Department of Neurosurgery
§Walton Center NHS Foundation Trust, Liverpool, United Kingdom
•Institute of Psychology Health and Society, University of Liverpool, Liverpool, United Kingdom.

**Email address**

Nisaharan Srikandarajah- nishsri@liv.ac.uk

Martin Wilby- martin.wilby@thewaltoncentre.nhs.uk

Simon Clark- simon.clark@thewaltoncentre.nhs.uk

Adam Noble- adam.noble@liv.ac.uk

Paula Williamson- prw@liv.ac.uk

Tony Marson- marjon01@liv.ac.uk

**Keywords**

Cauda Equina Syndrome, Surgery, Outcomes, Core Outcome Set, Systematic Literature Review, Qualitative Interviews, Delphi Survey, Consensus Meeting

**Corresponding Author**

Mr Nisaharan Srikandarajah, Spinal Research Team, Room 2:29, Clinical Sciences Center, University of Liverpool, Lower Lane, Liverpool, UK, L9 7LJ

E-mail: nishsri@liv.ac.uk

Telephone: 01515295945



For peer review only - http://bmjopen.bmj.com/site/about/guidelines.xhtml

**Word Count 5,246**


## ABSTRACT

### Introduction

Cauda Equina Syndrome (CES) is a serious neurological condition most commonly due to compression of the lumbosacral nerve roots, which can result in significant disability. The evidence for acute intervention in CES is mainly from retrospective studies. There is heterogeneity in the outcomes chosen for analysis in these studies, which makes it difficult to synthesise the data across studies. This study will develop a core outcome set for use in future studies of CES, engaging with key stakeholders and using transparent methodology. This will help ensure that relevant outcomes are used in future, and will facilitate attempts to summarise data across studies in systematic reviews.

### Methods and Analysis

A systematic literature review will document all the outcomes for CES after surgery mentioned in the literature. The qualitative interviews will be semi structured, audio recorded, transcribed and thematically analysed with the use of NVivo version 10 to determine the themes and the outcomes described by CES patients. The outcomes from the literature review and patient interviews will be combined and prioritised to determine what the most important outcomes are in CES research studies to patients and healthcare professionals. The prioritisation will be done through a two-round iterative Delphi survey and a consensus meeting. This process will decide the core outcome set for patients with CES.

### Ethics and Dissemination

REC and HRA approval was obtained on the 6/12/16 for the qualitative interviews from South Central - Hampshire A REC. REC reference 16/SC/0587. REC and HRA approval was obtained on 26/3/18 for the Delphi process and consensus meeting from North West- Greater Manchester Central REC. REC reference was 18/NW/0022. The final core outcome set will be published and freely available.


For peer review only

**Registration Details**

This study is registered with the Core Outcome Measures in Effectiveness Trials (COMET) database as study 824.

**Strengths and Limitations of the Study**

- The main strength for this study is a transparent consensus process which involves key stakeholders (patients and healthcare professionals) to decide a core outcome set for CES.
- A core outcome set will allow synthesis of data from future CES research studies and allow an evidence based treatment and management plan to be developed.
- The development of a core outcome set relies on the assumption that the key stakeholders will eventually come to a consensus.
- The outcomes that constitute the "core outcome set" will be reported. How these outcomes will be measured will not be determined in this study and requires further work.

## INTRODUCTION

Cauda equina syndrome (CES) is due to dysfunction of the lumbosacral nerve roots beneath the conus medullaris resulting in sensory-motor deficits of the lower limbs and sphincter dysfunction. Symptoms and signs include low back pain, unilateral or bilateral sciatica, saddle anaesthesia and motor weakness of the lower extremities with bladder and/or bowel dysfunction [1 2]. The most common cause of CES is a herniated lumbar disc, and represents 2% of all herniated lumbar discs. CES has an incidence of 2 per 100,000 in England and is an indication for emergency decompression surgery [3 4 5]. Other less common etiologies include spinal stenosis, spinal tumours, hematomas, fractures, and infections [2]. The National Spinal Task Force showed that there are 981 operations done each year for CES in the UK from 2010 to 2011 [6]. Surgical intervention for CES is not a rare procedure and the economic burden of severe disability is a worrying unknown for both patient quality of life and development of appropriate health services.

For peer review only

The evidence for acute intervention in CES is mainly from retrospective studies[7 8]. The importance of categorising CES into CES incomplete (CESI) and CES complete with urinary retention (CESR) has been highlighted in the literature[4]. CESR describes painless urinary retention with overflow incontinence and complete perianal sensory loss. When the patient complains of CESI, the symptoms include urinary issues of neurogenic origin including loss of desire to void, altered urinary sensation, and hesitancy with partial saddle anaesthesia.

It is documented in the literature that timely operative decompression for CES secondary to herniated lumbar disc can lead to improved outcomes in patients [9 7 8]. In fact, delay or missed diagnosis of this condition incurs heavy litigation costs to the NHS at £336,000 (US $549,427) per case on average [10] as reported to the Medical Defence Union in the UK.

**Rationale for the development of a "Core Outcome Set" (COS)**

An "outcome" in relation to clinical research studies is defined to be a measurement or observation used to capture and assess the effect of treatment such as assessment of the side effects (risk) or effectiveness (benefits). [11]

Before the systematic literature review a scoping review was undertaken[12]. It was identified that there were no randomised controlled trials, many retrospective observational studies and few prospective studies reporting the clinical outcome of patients with CES. There is heterogeneity and inconsistency in the outcomes reported in the literature for CES. The outcomes reported in the literature have not been independently validated as important to key stakeholders.

There is no defined core outcome set in cauda equina syndrome (CES) currently and this protocol will describe the methods of how to develop it. A core outcome set defines the minimum outcomes that should be consistently measured and reported in clinical trials in a specific area of healthcare [13]. With this there will be greater reporting consistency and a reduction in outcome reporting bias in healthcare studies contributing to systematic reviews and meta-analysis [14] that can lead to informed healthcare decisions.

For peer review only

Initially, a systematic literature review and qualitative patient interviews will be conducted to document the outcomes for CES patients after surgery. These outcomes will be combined and prioritised through two rounds of a Delphi process with key stakeholders and a consensus meeting to decide the core outcome set. The core outcome set would be published and used for future research studies and improving outcome reporting in CES.

The development of core outcome sets has been done successfully in rheumatology with the OMERACT group (Outcomes Measures in Rheumatoid Arthritis Clinical Trials). This international collaboration was developed in the early 1990s involving patients in the development of core outcome sets and has improved consistency of reported trials in this speciality [14 15]. The Core Outcome Measures in Effectiveness Trials (COMET) initiative advocates the involvement of patients and currently holds a database of on-going core outcome set developers[16] to minimise duplication and foster health service user engagement [13 17].

**Scope of the COS**

We aim to identify "what" outcomes of patients with CES are of concern to key stakeholders using transparent methodology. We are not intending to consider how these outcomes should be measured. The 11 minimum Core Outcome Set Standards for Development (COS-STAD) recommendations are addressed in this protocol [18] (**Table 1**).

**Table 1. COS-STAD recommendations**

| Domain | Standard Number | Methodology | Notes |
|---|---|---|---|
| Scope Specification | 1 | The research or practice setting in which the COS is to be applied | Research studies that will inform clinical decision making |
| | 2 | The health condition(s) covered by the COS | All severities of Cauda Equina Syndrome |
| | 3 | The population(s) covered by the COS | Human adults aged 18 or above |
| | 4 | The intervention(s) covered by the COS | Clinical management of CES including surgery |
| Stakeholders | 5 | Those who will use the COS in research | Clinical trialists in CES are healthcare |

For peer review only

| | | | |
|---|---|---|---|
| involved | | | professionals who manage CES patients. They are included in standard 6. |
| | 6 | Healthcare professionals with experience of patients with the condition | This will include clinicians, experts and healthcare professionals involved in CES management |
| | 7 | Patients with the condition or their representatives | Patients with a diagnosis of CES will be included [19] |
| Consensus Process | 8 | The initial list of outcomes considered both healthcare professionals and patients views | Systematic Literature review [20] considered healthcare professional views. Qualitative interviews considered patient views. |
| | 9 | A scoring process and consensus definition were described a priori | Described in “Scoring” and “Analysis” section of this protocol |
| | 10 | Criteria for including/dropping/adding outcomes were described a priori | Described in “Analysis” section of this protocol |
| | 11 | Care was taken to avoid ambiguity of language used in the list of outcomes | Plain language and clinical explanations available. These will be pilot tested with patients and healthcare professionals. |

**Registration**

The study is registered on the COMET database as study 824 (http://www.comet-initiative.org/studies/details/824?result=true).

For peer review only

## METHODS AND ANALYSIS

Development of the core outcome set will be developed in four phases with their estimated time frames highlighted in the overall study timeline (**Figure 1)**. Timeframes includes the estimated duration for ethical approval, study recruitment and analysis.

### *Phase 1: Systematic Literature Review*

#### Research Question

What outcomes are reported in the medical literature after surgery for CES?

#### Summary

The aim of the systematic literature review was to summarise the reporting standards of the clinical outcomes after surgery in CES patients following the PRISMA guidelines [21]. Most CES cases are due to lumbar disc herniation [22], which requires urgent surgical intervention. Study inclusion was limited to articles with patients who were surgically managed and whose outcomes were recorded.

The systematic literature review summarised the outcomes that had been mentioned in the literature and categorised them into a known taxonomy [23]. 1873 articles were identified through the search strategy of which 61 met the inclusion criteria. Inclusion criteria specified details regarding the study design, diagnosis, procedure, publication date, language and the patient age. 737 outcomes were reported verbatim in the 61 included articles. These were then



For peer review only - http://bmjopen.bmj.com/site/about/guidelines.xhtml

categorised to 20 higher order groupings called “outcome domains.” The most commonly reported outcomes were bladder function (70.5%), motor function (63.9%), and sensation (50.8%). There was significant variation in the terms used for each outcome for example, bladder function outcome domain had 141 different terms. Significant heterogeneity was evident in the outcomes reported in CES research studies. This highlighted a need for a core outcome set in CES to be developed. [20]

***Phase 2: Qualitative Interviews***

**Research Question**

What outcomes have CES patients experienced after surgery and how do they feel about the management before and after surgery?

**Method**

The objectives of the qualitative interviews with CES patients are:

- To explore the patient experience of living with CES.
- To document what the patient describes as the most important outcomes they are experiencing.
- To determine what service improvements can be made to improve CES management and aftercare.
- To determine who should be the key stakeholders in the Delphi survey.
- Identify appropriate language to use for the Delphi survey [24].

These interviews will be documented with audio recorded transcripts. The list of all potential outcomes from the systematic review and qualitative interviews will be placed into outcome domains by the research team to avoid repetition by qualitative method of content analysis [25]. The qualitative interviews will be piloted with 2 CES patients to establish if the interview structure and technique is clear, understandable, and capable of answering the research

For peer review only

questions. This would recognise any corrections that need to be made to the interview structure or technique. Inclusion and exclusion criteria are shown in **Table 2**.

**Table 2**. Inclusion and Exclusion criteria for qualitative interviews

| **INCLUSION CRITERIA** | **EXCLUSION CRITERIA** |
|---|---|
| Adult patients | Adults unable to consent for research |
| Diagnosis of Cauda Equina Syndrome | |
| Patient underwent a surgical procedure for CES | |
| Less than 10 years since the surgical procedure | |
| Ability to converse in English and to consent for research | |

**Participant Selection**

Adult patients for the qualitative interviews will be selected from those coded as having a diagnosis of cauda equina syndrome in the medical records. There is an existing database of cauda equina patients who have been operated on and followed up by consultants, registrars or nurse specialists depending on the next available clinic. Adult patients will be 18 years or older who have had spinal surgery to remove the compressive lesion at a single tertiary NHS institution over the past 10 years. The qualitative interviews will capture short and long term outcomes that are deemed important to them. Duration of the recorded outcomes will be calculated since the initial operation for CES.

Stratified purposive sampling [26] was chosen in which the aim is to select groups that display variation in particular characteristics so the subgroups can then be compared. Characteristics known to have an impact on the outcomes being investigated have been identified- severity of CES (CESI or CESR) [27] then there is a subgroup about which little is known and whose circumstances and views need to be explored; short (≤2 years) or long term (>2 years and ≤10 years) since the operation (see **Table 3**). This will produce 4 subcategories to populate. This is to prevent potential bias you may get from having many patients who presented with a severe



For peer review only - http://bmjopen.bmj.com/site/about/guidelines.xhtml

clinical picture and poor outcomes being more forthcoming and vocal. All subcategories for the sampling frame will be deemed a priority. Half the participants would ideally be male and half would be female.

**Table 3.** Sampling frame with suggested quotas

| | **CESI (Cauda Equina Syndrome Incomplete)** | **CESR (Cauda Equina Syndrome with retention)** |
|---|---|---|
| **Short term since the operation (≤2 years)** | 10 participants | 10 participants |
| **Long term since the operation (>2 years ≤10 years)** | 10 participants | 10 participants |

There is an existing database of 200 patients with contact details and clinical details of presentation and management, which will be updated up to the current date to exclude patients who are deceased. This should produce 50 patients per category. Due to reasons such as long travel distance from institution, not interested in participating it is anticipated that up to 10 patients may reply from each category, which would produce up to 40 patients in total. Options will be given to be interviewed at home, via electronic media (Skype), over the phone or to attend the hospital in person. After informed consent, patients will be interviewed until “data saturation” is reached. The research team will decide when data saturation is reached. Data saturation is the point where increasing the sample size no longer contributes to new evidence [28] moreover even large qualitative studies do not interview more than 50 people [29]. Additional patients will be interviewed in the subcategories if one group has a better response rate until data saturation is achieved.

Sticking rigidly to a sample frame could be counter-intuitive as one patient can be data rich during the interview as opposed to interviewing 5 patients where data is not rich. The aim is to collect rich data to allow in depth analysis [28]. So, although the sampling frame may serve as a guide it will not be used to start restricting participants especially at the initial stages of doing the qualitative interviews until data saturation is achieved.



For peer review only - http://bmjopen.bmj.com/site/about/guidelines.xhtml

An information leaflet and stamped addressed envelope to return the response slip will be sent to participants with a consent form. Patients will have 3 weeks to “opt-out” of the study by returning a response slip, through email or telephone with the research team. After this the participants will receive a phone call from the research team to confirm interest for participating in the study, to answer any further questions and to arrange a time and location for the interview.

**Interview Format and Analysis**

A semi-structured interview format will be utilised as per our topic guide (**Supplementary file 1).** Qualitative semi-structured interviews were chosen over questionnaires and focus groups as it was believed that patient opinions over sensitive subject matter such as bowel, bladder and sexual function would be better elicited in a private one to one interview and they were less likely to inhibit their contribution [28]. In addition, one-to-one interviews are more accessible for potential participants and for patients with mobility restrictions.

Informed consent will be obtained prior to the interview where anonymity and confidentiality will be expressed. The consent will also request the patient’s permission for their general practitioner (GP) to be informed of their involvement in the study. This is so that if there is any distress during the patient interviews, which requires medical management they can be referred to their GP. Open-ended non-leading questions on their diagnosis, management post operatively in hospital and management in the community will be asked allowing the participant to describe their experiences without unnecessary interruption [29]. Discussion will be directed towards outcomes of importance to the patient as seen in the topic guide. The interviewer will not discuss their own opinions about CES and if these are asked they will be answered at the end of the interview session. Reflexivity is an important concept during qualitative research for striving towards objectivity and neutrality[28] and the analysis of the interviews will consider if bias from the interviewer’s own beliefs may have crept in. It is anticipated that the interview will last for 45 minutes to an hour at each sitting to prevent the participant feeling fatigued. The same interviewer (NS) will be used for all the patient interviews. All interviewees will be made aware that the interviewer is a doctor not involved in



For peer review only - http://bmjopen.bmj.com/site/about/guidelines.xhtml

their on-going care. A sample of the transcripts will be reviewed by a supervisor not involved in the qualitative interviews to confirm that they were undertaken in a satisfactory manner. Initially, the transcripts will be reviewed to start identifying which outcomes are important to the patients by labelling the data using NVivo qualitative data analysis software version 10. A pragmatic approach will be taken by using thematic analysis as per the Braun and Clarke method[30]. It is a pattern-based qualitative method like grounded theory[31] and interpretative phenomenological analysis[32] but is not linked to a specific theoretical framework. This method will allow summarisation of the key outcomes of each individual transcript and overall themes whilst retaining the context and language in which it was expressed[28]. The qualitative interviews will be reported as outlined by the consolidated criteria for reporting qualitative research (COREQ); a 32 item checklist [33].

***Phase 3: The Delphi Survey***

The outcomes from the systematic literature review and qualitative interviews will create a long list[11]. This will be condensed by grouping similar outcomes into domains and conforming with the taxonomy used in the systematic literature review [20 23]. This will be reviewed and agreed by the study team and pilot tested with the key stakeholders before the Delphi survey is distributed.

**Research Question**

Which outcomes do patients and healthcare professionals think should be included in a core outcome set for patients with CES?

**Method**

All patients with CES will be invited to participate in the Delphi survey regardless of whether they had had surgery or not. Although there are a minority of participants in the category of non-operative management of CES[34] it was decided by the study team that including them will

For peer review only

be an opportunity to consider their input and maximise recruitment. The Delphi will be done by healthcare professionals and patients.

To achieve a priority list, we will use the "modified" Delphi method[35] as opposed to the "traditional" Delphi method[36]. Traditionally in a Delphi survey patients are asked open questions in the first round of the Delphi and the answers would constitute the outcomes rated in the second round. In the "modified" Delphi, which will be used in this study, rating the outcomes will take place over two rounds. A list of outcomes previously attained from the systematic literature review and qualitative interviews will be presented in the first round of the Delphi [35]. Patients can also suggest outcomes that have not been mentioned in the first round but these will not be scored. They will be considered for inclusion into the second round of the Delphi if, as judged by the CES study team, the outcome does not reflect or is not similar to another outcome already listed. The CES study team includes a patient representative.

The level of anonymity will be "fully anonymised" [37] so participants do not know the identities of other individuals in the group and they will not know the specific answers other individuals give. In round 2 of the Delphi, participants will know the group responses from the patient group and the healthcare professional group. Individual participants can decide to keep their original rating or to change their rating in the next round. This will lead to the group converging on a consensus opinion over the course of these two rounds [37].

**Inclusion criteria**

Participants will be recruited from two key stakeholder groups: patients and healthcare professionals. All participants will be adults over 18 years of age and able to complete an online survey in the English language.

*Patients*- Participants who have had an operation for CES.

*Healthcare Professionals*- All members of the clinical team involved in directly caring for a patient with CES such as:

- Spinal surgeons
- Spinal specialist nurses
- Neuro-rehabilitation doctors



For peer review only - http://bmjopen.bmj.com/site/about/guidelines.xhtml

**Sampling and Recruitment**

*Patients*- At the main site the clinical care team have a pre-existing database of CES patients they have clinically managed. The clinical care team will send an invitation letter to the home address of these patients. There will be no follow up calls or further correspondence. It is the patient's decision if they wish to be involved and the invitation will contain details of the website address patients can access if they wish to find out more details regarding the study. Online patient groups for CES will be contacted internationally. A named contact for each group will act as the liaison member to circulate the participant invitation email and poster. This may include the patient groups sharing the recruitment details on social media.

*Healthcare Professionals*- The main study site has spinal MDT (multi-disciplinary team) meetings held weekly. The coordinator has a pre-set mailing list that goes to healthcare professionals involved in the meeting. This will be used to send the participant invitation email. The membership of national and international associations will be contacted and invited to participate. They include different healthcare professionals in their membership categories. Some examples are listed below:

- Society of British Neurological Surgeons (SBNS)
- British Association of Spine Surgeons (BASS)
- World Federation of Neuro-rehabilitation (WFNR)
- Spinal Injuries Association (SIA)

Known contacts of the CES study group will be contacted and invited to participate. Snowballing sampling will be used to increase the sample size. The participant invitation email/ letter will be the first contact for healthcare professionals and patients, which is a short introduction and summary of the study. If they are interested further the participant can proceed to the registration website for further details and obtain a copy of the participant information leaflet.

**Sample Size**

There are no strict recommendations for the number of participants (patients and healthcare


For peer review only - http://bmjopen.bmj.com/site/about/guidelines.xhtml

professionals) required in a Delphi study to gain consensus [37]. In general, having more participants will increase the reliability of the group judgement [38]. A pragmatic approach to sample size will be taken and all individuals who meet the inclusion criteria as identified above will be invited to participate. The recruitment phase will be 2 months before the first round of the Delphi survey is released. Documentation of the organisations who distribute the Delphi invitation from each stakeholder group will be recorded. No further participants will be invited after the first round of the Delphi.

**Consent**

Consent will be implicit by the participant (patients and healthcare professionals) registering their name and email address to take part in the Delphi survey via the website.

**Questionnaires**

The questionnaire is constructed and delivered in an online format using the DelphiManager software developed by the COMET initiative. Before starting the questionnaire, the participant will be asked to clarify which of the two stakeholder groups they belong to. For each stakeholder group, specific information will be collected:

- *Patients*- Age, gender, location, surgery for CES- yes/no, years since surgery for CES, employed- full time/ employed- part time/ unemployed
- *Healthcare professionals*- Practicing Field (spinal surgeon, specialist nurse, neuro-rehabilitation etc), years in practice, location, gender

Following confirmation of their eligibility to participate in the study, participants will be sent an on-line link to access the first round of the Delphi process. Instructions of how to complete the questionnaire will be included at the beginning of each round. Only participants who respond to the 1st round of the Delphi will be invited to participate in 2nd round taking the assumption that if they had not participated in the first round they would be unwilling to participate in the second round. Data will be collected over at least a 4-week period for each round of the Delphi process. Participants who have not completed the survey will be sent reminders via email when they have 2 weeks, 1 week and 48 hours remaining for completion of the survey. Participants

For peer review only

who have not completed the questionnaire within 4 weeks of the start will be deemed not to have completed that round of the Delphi. The language used by patients in the qualitative interviews will be used to help term the outcomes for the Delphi. Plain language summaries by the COMET Patient Participation, Involvement and Engagement (PoPPIE) group was used to develop the Delphi information sheet. The Delphi will be piloted with 2 participants from each stakeholder group to highlight any issues with understanding or validity.

**Scoring**

For an outcome to be included in the core outcome set there must be a majority agreement of the critical importance of the outcome and minority agreement that the outcome is not important [39]. This is in par with the GRADE (Grading of Recommendations Assessment, Development and Evaluation) working group recommendations (http://www.gradeworkinggroup.org; [40 41]. At the beginning of the Delphi, participants will be reminded the importance of completing the entire Delphi process. Round one of the Delphi study we will ask participants to rate each outcome using a 9 point Likert scale. This scoring system was chosen after previous studies and expert databases showed it differentiates the most between questionnaire items [16 37]. 7-9 indicates critical importance. 4 to 6 represents outcomes that are important but not critical whilst 1 to 3 are deemed to be of limited importance. All outcomes will be carried through to second round with anonymised feedback of first round scores from the patient group and from the healthcare professional group displayed for each outcome. The feedback will show the cumulated scores from each stakeholder group for each outcome and the participant will be asked to rate the outcomes again using the same 9 point Likert scale. If they change their score on the second round they will have the opportunity to explain their reasoning for this. Outcomes which have been suggested in round 1 by the participants and deemed appropriate by the study group will then be entered in for rating in the second round by key stakeholders. After the final Delphi round, there will be a list developed from all stakeholder groups, which will be submitted to a face to face consensus meeting of key stakeholders to discuss what outcomes that should be finally included in the core outcome set. All participants who had completed both rounds of the Delphi survey will be



For peer review only - http://bmjopen.bmj.com/site/about/guidelines.xhtml

eligible for invitation to the consensus meeting. A trained independent facilitator would chair this meeting.

**Analysis**

Consensus that an outcome should be included in the core outcome set is defined as 70% or more scoring it as 7 to 9 and fewer than 15% scoring it as 1 to 3, which is has been seen to be successful with the development of other core outcome sets [42 43] (**Table 4**). This will be done for each stakeholder group. Results at the multiple rounds of the Delphi process and consensus meeting will be documented to include the number of participants invited, number completing the section, measure of each group response to an outcome leading to a comprehensive list of all outcomes that should be included in the COS CES.

**Table 4**. Definitions of a consensus

| **Classification of consensus** | **Description** | **Definition** |
|---|---|---|
| **IN** | Consensus that outcome should be included in the core outcome set | 70% or more participants scoring as 7 to 9 AND <15% participants scoring as 1 to 3 in both stakeholder groups |
| **OUT** | Consensus that outcome should not be included in the core outcome set | 50% or less participants scoring 7 to 9 in both stakeholder groups |
| **NO CONSENSUS** | Uncertainty about importance of outcome | Anything else |

**Attrition**

It is expected that some participants will drop out after each round of the Delphi. Each participant will be given a unique participant number when they complete the first round of the Delphi, which will allow calculation of the attrition rates between rounds. This will allow



For peer review only - http://bmjopen.bmj.com/site/about/guidelines.xhtml

identification of participants who have completed all rounds and see if there is any difference bias between those participants who complete the process. Mean round 1 scores for the participants who completed round 1 and round 2 will be compared with those that dropped out after round 1.

***Phase 4: Consensus Meeting***

All participants registering for the Delphi survey will be asked if they would be happy to attend a face to face consensus meeting involving patients and healthcare professionals. They will need to complete both rounds of the Delphi survey to be eligible to attend. This would be set up as a tick box on the registration page for the online Delphi.

40 participants will be invited to the consensus meeting. This will include 20 healthcare professionals and 20 patients. Out of the 40 participants; 30 will be from the UK and 10 will be international. Standard travel expenses and hotel accommodation will be reimbursed or provided. 10 of the participants at the consensus meeting will be invited before the Delphi survey is released to attend the consensus meeting but on the premise, that both rounds of the Delphi are completed. This is to make sure there is representation at the consensus meeting from key stakeholder organisations closely involved with CES patients, research or management. 30 participants at the consensus meeting will be those who have completed both rounds of the Delphi and ticked their interest to attend the consensus meeting during registration.

In the development of a breast reconstruction core outcome set patients and professionals were recruited in a 2:1 ratio so that patients' views were represented preferentially as the procedure is a patient selected optional intervention [44]. In our study, clinical intervention for cauda equina syndrome is performed as an emergency so it was deemed appropriate by the study team to have a 1:1 ratio of patients and healthcare professionals. This is to maximise the number of participants involved to help achieve consensus. In addition, the core outcome set should reflect all key stakeholders input equally. If there is an overwhelming response with

For peer review only

more than 40 participants interested in attending the consensus meeting the study team will apply stratified purposive sampling. On the day of the consensus meeting informed consent will be obtained from the patient participants.

Outcomes categorised as “consensus in” across both stakeholder groups from the Delphi survey **(Table 4)** will be included in the final core outcome set. Outcomes categorised as “consensus out” across both stakeholder groups from the Delphi survey will be excluded from the final core outcome set. Results of the Delphi survey will be discussed at the consensus meeting and the main discussion will be regarding the outcomes deemed as achieving “no consensus” in the Delphi survey. Participants at the meeting will vote on these outcomes. The same criteria for consensus used in the Delphi survey **(Table 4)** will be used in the consensus meeting. All outcomes that reach “consensus in” will be included in the core outcome set. All outcomes in the “consensus out” or “no consensus” category after voting in the consensus meeting will not be included in the core outcome set. If there is no agreed final core outcome set at the end of the first meeting subsequent meetings will be arranged for this to happen. The participants who had completed both rounds of the Delphi survey would be invited to attend another consensus meeting if required.

## PATIENT INVOLVEMENT

Patients will be involved in the design, review and recruitment of the study. The scope of the research question will be decided with the study team that includes 2 research partners who are patients with CES. The qualitative interviews will be trailed with the patient research partners and the topic guide will be reviewed by them. Pilot testing of the Delphi survey will be done by the patient research partners who will be asked to review the patient explanations of the outcomes and the questions on the registration page. Patients will be involved in the recruitment stage of the Delphi as they will be requested via social media to forward the website link for the Delphi survey to any relevant known contacts.

## ETHICS AND DISSEMINATION

For peer review only

REC and HRA approval was obtained on the 6 December 2016 for the qualitative interviews from South Central - Hampshire A Research Ethics Committee. REC reference 16/SC/0587. REC and HRA approval was obtained on 26 March 2018 for the Delphi process and consensus meeting from North West- Greater Manchester Central Research Ethics Committee. REC reference was 18/NW/0022. We intend to publish the results of the core outcome set for patients with CES in an open access journal. It will also be made available through the CES patient charity websites. Results will be disseminated through International and national presentations. The next step would be to identify the appropriate measurement instrument for each of the outcomes in the core outcome set [45]. Core outcome sets are developed in a number of clinical areas and their use is advocated in the UK by the National Institute for Health Research (NIHR) Health Technology Assessment (HTA), Cochrane Reviews of the effects of Healthcare intervention [46] and by World Health Organisation (WHO) handbook for guideline development [17]. The NIHR HTA has added this statement to their application form, “Where established core outcomes exist they should be included among the list of outcomes unless there is a good reason to do otherwise.” By developing the CES core outcome set we intend to reduce outcome reporting bias, heterogeneity, and improve the quality of research studies in CES. This will allow us to synthesise the data and make more robust evidence based decisions regarding the management of CES.

**Author Contributions**

NS, MW, SC conceived the project. TM is the principal investigator for the study. NS is the clinical research fellow responsible for management of the project, wrote the protocol and manuscript. TM, PW, AN, MW, SC provide supervision, have input in all aspects of the project, commented on drafts of the manuscript. All authors have read and approved the manuscript. Special thanks to Ms Claire Thornber and Mr Steven Smith as patient research partners in this study.

**Funding**



For peer review only - http://bmjopen.bmj.com/site/about/guidelines.xhtml

The paper is independent research funded by the Walton Centre Research Funds and Royal College of Surgeons Research Fellowship.

**Competing Interests**

None Declared

**Supplementary file 1**- Topic guide

**Figure 1.** The overall study timeline

**REFERENCES**

1. Kostuik JP. Controversies in cauda equina syndrome and lumbar disk herniation. *Current Opinion in Orthopaedics* 1993;4(2):125-28.
2. Gardner A, Gardner E, Morley T. Cauda equina syndrome: A review of the current clinical and medico-legal position. *Eur Spine J* 2011;20(5):690-97. doi: http://dx.doi.org/10.1007/s00586-010-1668-3
3. Gitelman A, Hishmeh S, Morelli BN, et al. Cauda equina syndrome: a comprehensive review. *Am J Orthop (Belle Mead NJ)* 2008;37(11):556-62.
4. Gleave JR, Macfarlane R. Cauda equina syndrome: what is the relationship between timing of surgery and outcome? *Br J Neurosurg* 2002;16(4):325-8.
5. Sorbie C. Cauda equina syndrome. *Orthopedics* 2009;32(6):397. doi: http://dx.doi.org/10.3928/01477447-20090511-31
6. Taskforce TNS. Commissioning Spinal Services - Getting The Service Back On Track . January 2013
7. Todd NV. Cauda equina syndrome: The timing of surgery probably does influence outcome. *Br J Neurosurg* 2005;19(4):301-06. doi: http://dx.doi.org/10.1080/02688690500305324
8. Srikandarajah N, Boissaud-Cooke MA, Clark S, et al. Does early surgical decompression in cauda equina syndrome improve bladder outcome? *Spine (03622436)* 2015;40(8):580-83. doi: 10.1097/BRS.0000000000000813
9. Ahn UM, Ahn NU, Buchowski JM, et al. Cauda equina syndrome secondary to lumbar disc herniation. *Spine* 2000;25(12):1515-22. doi: http://dx.doi.org/10.1097/00007632-200006150-00010
10. Daniels EW, Gordon Z, French K, et al. Review of medicolegal cases for cauda equina syndrome: what factors lead to an adverse outcome for the provider? *Orthopedics* 2012;35(3):200-00. doi: 10.3928/01477447-20120222-15
11. Williamson PR, Altman DG, Bagley H, et al. The COMET handbook: version 1.0. *Trials* 2017;18(3):280.



For peer review only - http://bmjopen.bmj.com/site/about/guidelines.xhtml

12. Armstrong R, Hall BJ, Doyle J, et al. 'Scoping the scope'of a cochrane review. *Journal of Public Health* 2011;33(1):147-50.
13. Williamson PR, Altman DG, Blazeby JM, et al. Developing core outcome sets for clinical trials: issues to consider. *Trials* 2012;13(1):132.
14. Kirkham JJ, Gargon E, Clarke M, et al. Can a core outcome set improve the quality of systematic reviews?–a survey of the Co-ordinating Editors of Cochrane Review Groups. *Trials* 2013;14(1):21.
15. Tugwell P, Boers M, Brooks P, et al. OMERACT: an international initiative to improve outcome measurement in rheumatology. *Trials* 2007;8(1):38.
16. COMET. The COMET Initiative. 2018
17. Williamson PR, Altman DG, Blazeby JM, et al. The COMET (core outcome measures in effectiveness trials) initiative. *Trials* 2011;12(Suppl 1):A70.
18. Kirkham JJ, Davis K, Altman DG, et al. Core Outcome Set-STAndards for Development: The COS-STAD recommendations. *PLoS medicine* 2017;14(11):e1002447.
19. Young B, Bagley H. Including patients in core outcome set development: issues to consider based on three workshops with around 100 international delegates. *Research Involvement and Engagement* 2016;2(1):25.
20. Srikandarajah N, Wilby M, Clark S, et al. Outcomes reported after surgery for Cauda Equina Syndrome: A Systematic Literature Review. *Spine* 2018
21. Moher D, Liberati A, Tetzlaff J, et al. Preferred reporting items for systematic reviews and meta-analyses: the PRISMA statement. *PLoS medicine* 2009;6(7):e1000097.
22. Kostuik JP. Medicolegal consequences of cauda equina syndrome: an overview. *Neurosurg* 2004;16(6):e8.
23. Dodd S, Clarke M, Becker L, et al. A taxonomy has been developed for outcomes in medical research to help improve knowledge discovery. *Journal of clinical epidemiology* 2018;96:84-92.
24. Keeley T, Khan H, Pinfold V, et al. Core outcome sets for use in effectiveness trials involving people with bipolar and schizophrenia in a community-based setting (PARTNERS2): study protocol for the development of two core outcome sets. *Trials* 2015;16(1):47.
25. Elo S, Kyngäs H. The qualitative content analysis process. *Journal of advanced nursing* 2008;62(1):107-15.
26. Quinn Patton M. Qualitative Research John Wiley & Sons: Ltd, 2005.
27. Gleave JRW, Macfarlane R. Cauda equina syndrome: What is the relationship between timing of surgery and outcome? *Br J Neurosurg* 2002;16(4):325-28. doi: http://dx.doi.org/10.1080/02688690210000032887
28. Ritchie J, Lewis J, Nicholls CM, et al. Qualitative research practice: A guide for social science students and researchers: Sage 2013.
29. Britten N. Qualitative research: qualitative interviews in medical research. *Bmj* 1995;311(6999):251-53.
30. Braun V, Clarke V. Using thematic analysis in psychology. *Qualitative research in psychology* 2006;3(2):77-101.
31. Charmaz K. Constructing grounded theory: A practical guide through qualitative analysis: Sage 2006.
32. Smith JA, Flowers P, Larkin M. Interpretative phenomenological analysis: Theory, method and research2009.

For peer review only

33. Tong A, Sainsbury P, Craig J. Consolidated criteria for reporting qualitative research (COREQ): a 32-item checklist for interviews and focus groups. *International journal for quality in health care* 2007;19(6):349-57.
34. Khurana R, Arora SK, Hemal A, et al. Successful non-operative management of cauda equina syndrome in a case of thalassemia major. *Pediatric Hematology Oncology Journal* 2016;1(2):41-43.
35. Keeley T, Williamson P, Callery P, et al. The use of qualitative methods to inform Delphi surveys in core outcome set development. *Trials* 2016;17(1):230.
36. Hasson F, Keeney S, McKenna H. Research guidelines for the Delphi survey technique. *Journal of advanced nursing* 2000;32(4):1008-15.
37. Sinha IP, Smyth RL, Williamson PR. Using the Delphi technique to determine which outcomes to measure in clinical trials: recommendations for the future based on a systematic review of existing studies. *PLoS medicine* 2011;8(1):e1000393.
38. Murphy M. Consensus development methods and their use in clinical guideline development. *Health Technol Assess* 1998;2:1-88.
39. Jaeschke R, Guyatt GH, Dellinger P, et al. Use of GRADE grid to reach decisions on clinical practice guidelines when consensus is elusive. *BMJ: British Medical Journal (Online)* 2008;337
40. Atkins D, Best D, Briss PA, et al. Grading quality of evidence and strength of recommendations. *BMJ (Clinical research ed)* 2004;328(7454):1490-90.
41. Guyatt GH, Oxman AD, Vist GE, et al. GRADE: an emerging consensus on rating quality of evidence and strength of recommendations. *BMJ (Clinical research ed)* 2008;336(7650):924-26.
42. Harman NL, Bruce IA, Callery P, et al. MOMENT–Management of Otitis Media with Effusion in Cleft Palate: protocol for a systematic review of the literature and identification of a core outcome set using a Delphi survey. *Trials* 2013;14(1):70.
43. Haywood K, Griffin X, Achten J, et al. Developing a core outcome set for hip fracture trials. *Bone Joint J* 2014;96(8):1016-23.
44. Potter S, Holcombe C, Ward J, et al. Development of a core outcome set for research and audit studies in reconstructive breast surgery. *British Journal of Surgery* 2015;102(11):1360-71.
45. Prinsen CA, Vohra S, Rose MR, et al. How to select outcome measurement instruments for outcomes included in a "Core Outcome Set"–a practical guideline. *Trials* 2016;17(1):449.
46. Rosenbaum SE, Glenton C, Oxman AD. Summary-of-findings tables in Cochrane reviews improved understanding and rapid retrieval of key information. *Journal of clinical epidemiology* 2010;63(6):620-26.

For peer review only



For peer review only - http://bmjopen.bmj.com/site/about/guidelines.xhtml

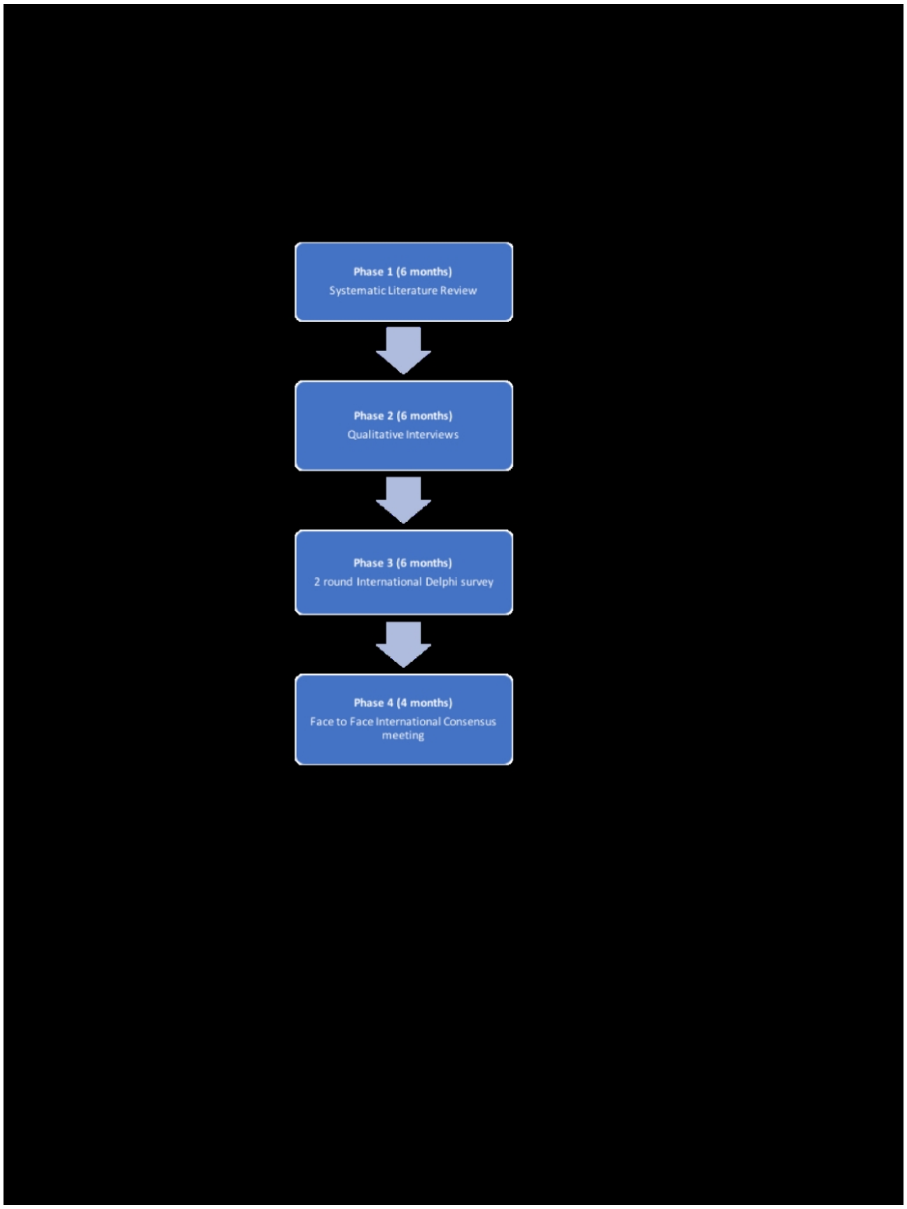


Figure 1. The overall study timeline

IRAS: 201946 Version: 1.1 Date: 23/09/16

# TOPIC GUIDE CES QUALITATIVE INTERVIEWS

## Aims and Objectives

-To explore the patient experience of living with Cauda Equina Syndrome (CES)
-To ascertain what the patient feels are the most important outcomes that they are experiencing
-To ascertain what outcomes the patient feels are the most important to research in to improve CES management and aftercare
-To determine who should be key stakeholders
-Identify appropriate language to use for patient Delphi iterative process.

## Introduction (5-10 mins)

Interviewer Name
Interviewer Occupation
Explain basic definition of CES
Explain looking for challenges experienced after the operation for CES
Explain expected intention, sensitive subjects and duration of interview and confidentiality
Confirm consent to qualitative interview

## Background (<5 mins)

Interviewee name
Interviewee age
Interviewee occupation
Other medical conditions
When was your operation for CES?

## Interview questions (30 mins)

How has your experience of this condition; Cauda Equina Syndrome been?
- What was it like before the back operation?
- What was it like after the back operation?

How do you feel your condition has been managed in hospital and in the community?

What were your expectations of life health-wise after the operation and what is the reality like?

For peer review only

Due to this condition what do you feel are the challenges to your health and wellbeing?
-bowel/bladder
-sex life
-back/ leg pain
-psychological
-anxiety/fear
-other

Would you be able to prioritise the importance of these for you now?

Was the importance of these different at earlier stages of the condition? (More relevant to those in the long term CES category)

Through this process of living with CES who else do you think has a good handle on the condition? If anyone? -Gauge other potential key stakeholders

Tell me a bit about the support you had for the condition?

**Closing remarks (5 mins)**

Considering your hospital, post op and follow up experience what would you have liked to change?
-support services
-more streamlined service with dedicated clinics
-research into timing for CES operations
-follow up as to the effects of long term CES

Offer the opportunity for the participant to comment on their interview transcript after transcription.

For peer review only

# PROTOCOL FOR THE DEVELOPMENT OF A CORE OUTCOME SET FOR CAUDA EQUINA SYNDROME: SYSTEMATIC LITERATURE REVIEW, QUALITATIVE INTERVIEWS, DELPHI SURVEY AND CONSENSUS MEETING


| | |
|---|---|
| Journal: | *BMJ Open* |
| Manuscript ID | bmjopen-2018-024002.R2 |
| Article Type: | Protocol |
| Date Submitted by the Author: | 08-Feb-2019 |


| | |
|---|---|
| Complete List of Authors: | Srikandarajah, Nisaharan; University of Liverpool, Institute of Translational Medicine<br>Noble, Adam; University of Liverpool, Institute of Psychology Health and Society<br>Wilby, Martin; The Walton Centre NHS Foundation Trust, Neurosurgery<br>Clark, Simon; The Walton Centre NHS Foundation Trust, Neurosurgery<br>Williamson, Paula; University of Liverpool , Institute of Translational Medicine<br>Marson, Anthony; University of Liverpool, Institute of Translational Medicine |


| | |
|---|---|
| <b>Primary Subject Heading</b>: | Neurology |
| Secondary Subject Heading: | Medical management, Patient-centred medicine, Qualitative research, Research methods, Surgery |
| Keywords: | Cauda Equina Syndrome, Core Outcome Set, Delphi, Systematic Literature Review, Qualitative Interviews, Consensus |
| | |


SCHOLARONE™ Manuscripts

# PROTOCOL FOR THE DEVELOPMENT OF A CORE OUTCOME SET FOR CAUDA EQUINA SYNDROME: SYSTEMATIC LITERATURE REVIEW, QUALITATIVE INTERVIEWS, DELPHI SURVEY AND CONSENSUS MEETING

**Nisaharan Srikandarajah*, Adam J Noble•, Martin Wilby§, Simon Clark§, Paula R Williamson*, Anthony Guy Marson***

**Institute**

*Institute of Translational Medicine, University of Liverpool, Liverpool, United Kingdom; Department of Neurosurgery
§Walton Center NHS Foundation Trust, Liverpool, United Kingdom
•Institute of Psychology Health and Society, University of Liverpool, Liverpool, United Kingdom.

**Email address**

Nisaharan Srikandarajah- nishsri09@gmail.com

Martin Wilby- martin.wilby@thewaltoncentre.nhs.uk

Simon Clark- simon.clark@thewaltoncentre.nhs.uk

Adam Noble- adam.noble@liv.ac.uk

Paula Williamson- prw@liv.ac.uk

Tony Marson- marjon01@liv.ac.uk

For peer review only

**Keywords**

Cauda Equina Syndrome, Surgery, Outcomes, Core Outcome Set, Systematic Literature Review, Qualitative Interviews, Delphi Survey, Consensus Meeting

**Corresponding Author**

Mr Nisaharan Srikandarajah, Spinal Research Team, Room 2:29, Clinical Sciences Center, University of Liverpool, Lower Lane, Liverpool, UK, L9 7LJ

E-mail: nishsri09@gmail.com

Telephone: 01515295945

**Word Count 5,480**



For peer review only - http://bmjopen.bmj.com/site/about/guidelines.xhtml

**ABSTRACT**

**Introduction**

Cauda Equina Syndrome (CES) is a serious neurological condition most commonly due to compression of the lumbosacral nerve roots, which can result in significant disability. The evidence for acute intervention in CES is mainly from retrospective studies. There is heterogeneity in the outcomes chosen for analysis in these studies, which makes it difficult to synthesise the data across studies. This study will develop a core outcome set for use in future studies of CES, engaging with key stakeholders and using transparent methodology. This will help ensure that relevant outcomes are used in future, and will facilitate attempts to summarise data across studies in systematic reviews.

**Methods and Analysis**

A systematic literature review will document all the outcomes for CES after surgery mentioned in the literature. The qualitative interviews with CES patients will be semi structured, audio recorded, transcribed and thematically analysed with the use of NVivo version 10 to identify outcomes and determine the themes described. The outcomes from the literature review and patient interviews will be combined and prioritised to determine what the most important outcomes are in CES research studies to patients and healthcare professionals. The prioritisation will be done through a two-round iterative Delphi survey and a consensus meeting. This process will decide the core outcome set for patients with CES.

**Ethics and Dissemination**

REC and HRA approval was obtained on the 6/12/16 for the qualitative interviews from South Central - Hampshire A REC. REC reference 16/SC/0587. REC and HRA approval was obtained on 26/3/18 for the Delphi process and consensus meeting from North West-Greater Manchester Central REC. REC reference was 18/NW/0022. The final core outcome set will be published and freely available.

**Registration Details**

This study is registered with the Core Outcome Measures in Effectiveness Trials (COMET) database as study 824.

For peer review only



For peer review only - http://bmjopen.bmj.com/site/about/guidelines.xhtml

**Strengths and Limitations of the Study**

- A systematic literature review following PRISMA guidelines will identify outcomes in the existing literature for Cauda Equina Syndrome (CES).
- Semi-structured qualitative interviews using a sampling frame to select a varied sample of CES patients will identify outcomes important to them.
- The consensus process of an international online Delphi survey and an international face to face consensus meeting will involve patients and healthcare professionals.
- A core outcome set will allow future CES research studies to use outcomes relevant to key stakeholders and allow synthesis of data in CES.
- The outcomes that constitute the core outcome set will be reported. "How" these outcomes are measured will not be determined in this study and requires further work.

## INTRODUCTION

Cauda equina syndrome (CES) is due to dysfunction of the lumbosacral nerve roots beneath the conus medullaris resulting in sensory-motor deficits of the lower limbs and sphincter dysfunction. Symptoms and signs include low back pain, unilateral or bilateral sciatica, saddle anaesthesia and motor weakness of the lower extremities with bladder and/or bowel dysfunction [1 2]. The most common cause of CES is a herniated lumbar disc, and represents 2% of all herniated lumbar discs. CES has an incidence of 2 per 100,000 in England and is an indication for emergency decompression surgery [3 4 5]. Other less common etiologies include spinal stenosis, spinal tumours, hematomas, fractures, and infections [2]. The National Spinal Task Force showed that there are 981 operations done each year for CES in the UK from 2010 to 2011 [6]. Surgical intervention for CES is not a rare procedure and the economic burden of severe disability is a worrying unknown for both patient quality of life and development of appropriate health services.

The evidence for acute intervention in CES is mainly from retrospective studies[7 8]. The importance of categorising CES into CES incomplete (CESI) and CES complete with urinary retention (CESR) has been highlighted in the literature[4]. CESR describes painless urinary

For peer review only - http://bmjopen.bmj.com/site/about/guidelines.xhtml

retention with overflow incontinence and complete perianal sensory loss. When the patient complains of CESI, the symptoms include urinary issues of neurogenic origin including loss of desire to void, altered urinary sensation, and hesitancy with partial saddle anaesthesia.

It is documented in the literature that timely operative decompression for CES secondary to herniated lumbar disc can lead to improved outcomes in patients [9 7 8]. In fact, delay or missed diagnosis of this condition incurs heavy litigation costs to the NHS at £336,000 (US $549,427) per case on average [10] as reported to the Medical Defence Union in the UK.

## Rationale for the development of a "Core Outcome Set" (COS)

An "outcome" in relation to clinical research studies is defined to be a measurement or observation used to capture and assess the effect of treatment such as assessment of the side effects (risk) or effectiveness (benefits). [11]

Before the systematic literature review a scoping review was undertaken[12]. It was identified that there were no randomised controlled trials, many retrospective observational studies and few prospective studies reporting the clinical outcome of patients with CES. There is heterogeneity and inconsistency in the outcomes reported in the literature for CES. The outcomes reported in the literature have not been independently validated as important to key stakeholders.

There is no defined core outcome set in cauda equina syndrome (CES) currently and this protocol will  describe the methods of how to develop it. A core outcome set defines the minimum outcomes that should be consistently measured and reported in clinical trials in a specific area of healthcare [13]. With this there will be greater reporting consistency and a reduction in outcome reporting bias in healthcare studies contributing to systematic reviews and meta-analysis [14] that can lead to informed healthcare decisions.

Initially, a systematic literature review and qualitative patient interviews will be conducted to document the outcomes for CES patients after surgery. These outcomes will be combined and prioritised through two rounds of a Delphi process with key stakeholders and a consensus meeting to decide the core outcome set. The core outcome set would be published and used for future research studies and improving outcome reporting in CES.

For peer review only

The development of core outcome sets has been done successfully in rheumatology with the OMERACT group (Outcomes Measures in Rheumatoid Arthritis Clinical Trials). This international collaboration was developed in the early 1990s involving patients in the development of core outcome sets and has improved consistency of reported trials in this speciality [14 15]. The Core Outcome Measures in Effectiveness Trials (COMET) initiative advocates the involvement of patients and currently holds a database of on-going core outcome set developers[16] to minimise duplication and foster health service user engagement [13 17].

**Scope of the COS**

We aim to identify "what" outcomes of patients with CES are of concern to key stakeholders using transparent methodology. We are not intending to consider how these outcomes should be measured. The 11 minimum Core Outcome Set Standards for Development (COS-STAD) recommendations are addressed in this protocol [18] (**Table 1**).

**Table 1. COS-STAD recommendations**

| **Domain** | **Standard Number** | **Methodology** | **Notes** |
|---|---|---|---|
| Scope Specification | 1 | The research or practice setting in which the COS is to be applied | Research studies that will inform clinical decision making |
| | 2 | The health condition(s) covered by the COS | All severities of Cauda Equina Syndrome |
| | 3 | The population(s) covered by the COS | Human adults aged 18 or above |
| | 4 | The intervention(s) covered by the COS | Clinical management of CES including surgery |
| Stakeholders involved | 5 | Those who will use the COS in research | Clinical trialists in CES are healthcare professionals who manage CES patients. They are included in standard 6. |
| | 6 | Healthcare professionals with experience of patients with the condition | This will include clinicians, experts and healthcare professionals involved in CES management |



For peer review only - http://bmjopen.bmj.com/site/about/guidelines.xhtml

| | | | |
|---|---|---|---|
| | 7 | Patients with the condition or their representatives | Patients with a diagnosis of CES will be included [19] |
| Consensus Process | 8 | The initial list of outcomes considered both healthcare professionals and patients views | Systematic Literature review [20] considered healthcare professional views. Qualitative interviews considered patient views. |
| | 9 | A scoring process and consensus definition were described a priori | Described in "Scoring" and "Analysis" section of this protocol |
| | 10 | Criteria for including/dropping/adding outcomes were described a priori | Described in "Analysis" section of this protocol |
| | 11 | Care was taken to avoid ambiguity of language used in the list of outcomes | Plain language and clinical explanations available. These will be pilot tested with patients and healthcare professionals. |

**Registration**

The study is registered on the COMET database as study 824 (http://www.comet-initiative.org/studies/details/824?result=true).

For peer review only

## METHODS AND ANALYSIS

Development of the core outcome set will be developed in four phases with their estimated time frames highlighted in the overall study timeline (**Figure 1)**. Timeframes includes the estimated duration for ethical approval, study recruitment and analysis.

### *Phase 1: Systematic Literature Review*

#### Research Question

What outcomes are reported in the medical literature after surgery for CES?

#### Summary

The aim of the systematic literature review was to summarise the reporting standards of the clinical outcomes after surgery in CES patients following the PRISMA guidelines [21]. Most CES cases are due to lumbar disc herniation [22], which requires urgent surgical intervention. Study inclusion was limited to articles with patients who were surgically managed and whose outcomes were recorded.

The systematic literature review summarised the outcomes that had been mentioned in the literature and categorised them into a known taxonomy [23]. 1873 articles were identified through the search strategy of which 61 met the inclusion criteria. Inclusion criteria specified details regarding the study design, diagnosis, procedure, publication date, language and the patient age. 737 outcomes were reported verbatim in the 61 included articles. These were then categorised to 20 higher order groupings called "outcome domains." The most commonly reported outcomes were bladder function (70.5%), motor function (63.9%), and sensation (50.8%). There was significant variation in the terms used for each outcome for example, bladder function outcome domain had 141 different terms. Significant heterogeneity was evident in the outcomes reported in CES research studies. This highlighted a need for a core outcome set in CES to be developed. [20]

### *Phase 2: Qualitative Interviews*

#### Research Question

For peer review only

What outcomes have CES patients experienced after surgery and how do they feel about the management before and after surgery?

**Method**

The objectives of the qualitative interviews with CES patients are:

- To explore the patient experience of living with CES.
- To document what the patient describes as the most important outcomes they are experiencing.
- To determine what service improvements can be made to improve CES management and aftercare.
- To determine who should be the key stakeholders in the Delphi survey.
- Identify appropriate language to use for the Delphi survey [24].

These interviews will be documented with audio recorded transcripts. The list of all potential outcomes from the systematic review and qualitative interviews will be placed into outcome domains by the research team to avoid repetition by qualitative method of content analysis [25]. The qualitative interviews will be piloted with 2 CES patients to establish if the interview structure and technique is clear, understandable, and capable of answering the research questions. This would recognise any corrections that need to be made to the interview structure or technique. Inclusion and exclusion criteria are shown in **Table 2**.

**Table 2**. Inclusion and Exclusion criteria for qualitative interviews

| **INCLUSION CRITERIA** | **EXCLUSION CRITERIA** |
|---|---|
| Adult patients | Adults unable to consent for research |
| Diagnosis of Cauda Equina Syndrome | |
| Patient underwent a surgical procedure for CES | |
| Less than 10 years since the surgical procedure | |
| Ability to converse in English and to consent for research | |

For peer review only

**Participant Selection**

Adult patients for the qualitative interviews will be selected from those coded as having a diagnosis of cauda equina syndrome in the medical records. There is an existing database of cauda equina patients who have been operated on and followed up by consultants, registrars or nurse specialists depending on the next available clinic. Adult patients will be 18 years or older who have had spinal surgery to remove the compressive lesion at a single tertiary NHS institution over the past 10 years. The qualitative interviews will capture short and long term outcomes that are deemed important to them. Duration of the recorded outcomes will be calculated since the initial operation for CES.

Stratified purposive sampling [26] was chosen in which the aim is to select groups that display variation in particular characteristics so the subgroups can then be compared. Characteristics known to have an impact on the outcomes being investigated have been identified- severity of CES (CESI or CESR) [27] then there is a subgroup about which little is known and whose circumstances and views need to be explored; short (≤2 years) or long term (>2 years and ≤10 years) since the operation (see **Table 3**). This will produce 4 subcategories to populate. This is to prevent potential bias you may get from having many patients who presented with a severe clinical picture and poor outcomes being more forthcoming and vocal. All subcategories for the sampling frame will be deemed a priority. Half the participants would ideally be male and half would be female.

**Table 3.** Sampling frame with suggested quotas

| | **CESI (Cauda Equina Syndrome Incomplete)** | **CESR (Cauda Equina Syndrome with retention)** |
|---|---|---|
| **Short term since the operation (≤2 years)** | 10 participants | 10 participants |
| **Long term since the operation (>2 years ≤10 years)** | 10 participants | 10 participants |

There is an existing database of 200 patients with contact details and clinical details of presentation and management, which will be updated up to the current date to exclude patients who are deceased. This should produce 50 patients per category. Due to reasons



For peer review only - http://bmjopen.bmj.com/site/about/guidelines.xhtml

such as long travel distance from institution, not interested in participating it is anticipated that up to 10 patients may reply from each category, which would produce up to 40 patients in total. Options will be given to be interviewed at home, via electronic media (Skype), over the phone or to attend the hospital in person. After informed consent, patients will be interviewed until "data saturation" is reached. The research team will decide when data saturation is reached. Data saturation is the point where increasing the sample size no longer contributes to new evidence [28] moreover even large qualitative studies do not interview more than 50 people [29]. Additional patients will be interviewed in the subcategories if one group has a better response rate until data saturation is achieved.

Sticking rigidly to a sample frame could be counter-intuitive as one patient can be data rich during the interview as opposed to interviewing 5 patients where data is not rich. The aim is to collect rich data to allow in depth analysis [28]. So, although the sampling frame may serve as a guide it will not be used to start restricting participants especially at the initial stages of doing the qualitative interviews until data saturation is achieved.

An information leaflet and stamped addressed envelope to return the response slip will be sent to participants with a consent form. Patients will have 3 weeks to "opt-out" of the study by returning a response slip, through email or telephone with the research team. After this the participants will receive a phone call from the research team to confirm interest for participating in the study, to answer any further questions and to arrange a time and location for the interview.

### Interview Format and Analysis

A semi-structured interview format will be utilised as per our topic guide (**Supplementary file 1).** Qualitative semi-structured interviews were chosen over questionnaires and focus groups as it was believed that patient opinions over sensitive subject matter such as bowel, bladder and sexual function would be better elicited in a private one to one interview and they were less likely to inhibit their contribution [28]. In addition, one-to-one interviews are more accessible for potential participants and for patients with mobility restrictions. Informed consent will be obtained prior to the interview where anonymity and confidentiality will be expressed. The consent will also request the patient's permission for their general practitioner (GP) to be informed of their involvement in the study. This is so



For peer review only - http://bmjopen.bmj.com/site/about/guidelines.xhtml

that if there is any distress during the patient interviews, which requires medical management they can be referred to their GP. Open-ended non-leading questions on their diagnosis, management post operatively in hospital and management in the community will be asked allowing the participant to describe their experiences without unnecessary interruption [29]. Discussion will be directed towards outcomes of importance to the patient as seen in the topic guide. The interviewer will not discuss their own opinions about CES and if these are asked they will be answered at the end of the interview session. Reflexivity is an important concept during qualitative research for striving towards objectivity and neutrality[28] and the analysis of the interviews will consider if bias from the interviewer's own beliefs may have crept in. It is anticipated that the interview will last for 45 minutes to an hour at each sitting to prevent the participant feeling fatigued. The same interviewer (NS) will be used for all the patient interviews. All interviewees will be made aware that the interviewer is a doctor not involved in their on-going care. A sample of the transcripts will be reviewed by a supervisor not involved in the qualitative interviews to confirm that they were undertaken in a satisfactory manner.

Initially, the transcripts will be reviewed to start identifying which outcomes are important to the patients by labelling the data using NVivo qualitative data analysis software version 10. A pragmatic approach will be taken by using thematic analysis as per the Braun and Clarke method[30]. It is a pattern-based qualitative method like grounded theory[31] and interpretative phenomenological analysis[32] but is not linked to a specific theoretical framework. This method will allow summarisation of the key outcomes of each individual transcript and overall themes whilst retaining the context and language in which it was expressed[28]. The qualitative interviews will be reported as outlined by the consolidated criteria for reporting qualitative research (COREQ); a 32 item checklist [33].

### *Phase 3: The Delphi Survey*

The outcomes from the systematic literature review and qualitative interviews will create a long list[11]. This will be condensed by grouping similar outcomes into domains and conforming with the taxonomy used in the systematic literature review [20 23]. This will be reviewed and agreed by the study team and pilot tested with the key stakeholders before the Delphi survey is distributed.



For peer review only - http://bmjopen.bmj.com/site/about/guidelines.xhtml

**Research Question**

Which outcomes do patients and healthcare professionals think should be included in a core outcome set for patients with CES?

**Method**

All patients with CES will be invited to participate in the Delphi survey regardless of whether they had had surgery or not. Although there are a minority of participants in the category of non-operative management of CES[34] it was decided by the study team that including them will be an opportunity to consider their input and maximise recruitment. The Delphi will be done by healthcare professionals and patients.

To achieve a priority list, we will use the "modified" Delphi method[35] as opposed to the "traditional" Delphi method[36]. Traditionally in a Delphi survey patients are asked open questions in the first round of the Delphi and the answers would constitute the outcomes rated in the second round. In the "modified" Delphi, which will be used in this study, rating the outcomes will take place over two rounds. A list of outcomes previously attained from the systematic literature review and qualitative interviews will be presented in the first round of the Delphi [35]. Patients can also suggest outcomes that have not been mentioned in the first round but these will not be scored. They will be considered for inclusion into the second round of the Delphi if, as judged by the CES study team, the outcome does not reflect or is not similar to another outcome already listed. The CES study team includes a patient representative.

The level of anonymity will be "fully anonymised" [37] so participants do not know the identities of other individuals in the group and they will not know the specific answers other individuals give. In round 2 of the Delphi, participants will know the group responses from the patient group and the healthcare professional group. Individual participants can decide to keep their original rating or to change their rating in the next round. This will lead to the group converging on a consensus opinion over the course of these two rounds [37].

**Inclusion criteria**

Participants will be recruited from two key stakeholder groups: patients and healthcare professionals. All participants will be adults over 18 years of age and able to complete an


For peer review only - http://bmjopen.bmj.com/site/about/guidelines.xhtml

online survey in the English language.

*Patients*- Participants who have had an operation for CES.

*Healthcare Professionals*- All members of the clinical team involved in directly caring for a patient with CES such as:

- Spinal surgeons
- Spinal specialist nurses
- Neuro-rehabilitation doctors

**Sampling and Recruitment**

*Patients*- At the main site the clinical care team have a pre-existing database of CES patients they have clinically managed. The clinical care team will send an invitation letter to the home address of these patients. There will be no follow up calls or further correspondence. It is the patient's decision if they wish to be involved and the invitation will contain details of the website address patients can access if they wish to find out more details regarding the study. Online patient groups for CES will be contacted internationally. A named contact for each group will act as the liaison member to circulate the participant invitation email and poster. This may include the patient groups sharing the recruitment details on social media.

*Healthcare Professionals*- The main study site has spinal MDT (multi-disciplinary team) meetings held weekly. The coordinator has a pre-set mailing list that goes to healthcare professionals involved in the meeting. This will be used to send the participant invitation email. The membership of national and international associations will be contacted and invited to participate. They include different healthcare professionals in their membership categories. Some examples are listed below:

- Society of British Neurological Surgeons (SBNS)
- British Association of Spine Surgeons (BASS)
- World Federation of Neuro-rehabilitation (WFNR)
- Spinal Injuries Association (SIA)

Known contacts of the CES study group will be contacted and invited to participate. Snowballing sampling will be used to increase the sample size. The participant invitation email/ letter will be the first contact for healthcare professionals and patients, which is a

For peer review only



For peer review only - http://bmjopen.bmj.com/site/about/guidelines.xhtml

short introduction and summary of the study. If they are interested further the participant can proceed to the registration website for further details and obtain a copy of the participant information leaflet.

**Sample Size**

There are no strict recommendations for the number of participants (patients and healthcare professionals) required in a Delphi study to gain consensus [37]. In general, having more participants will increase the reliability of the group judgement [38]. A pragmatic approach to sample size will be taken and all individuals who meet the inclusion criteria as identified above will be invited to participate. The recruitment phase will be 2 months before the first round of the Delphi survey is released. Documentation of the organisations who distribute the Delphi invitation from each stakeholder group will be recorded. No further participants will be invited after the first round of the Delphi.

**Consent**

Consent will be implicit by the participant (patients and healthcare professionals) registering their name and email address to take part in the Delphi survey via the website.

**Questionnaires**

The questionnaire is constructed and delivered in an online format using the DelphiManager software developed by the COMET initiative. Before starting the questionnaire, the participant will be asked to clarify which of the two stakeholder groups they belong to. For each stakeholder group, specific information will be collected:

- *Patients*- Age, gender, location, surgery for CES- yes/no, years since surgery for CES, employed- full time/ employed- part time/ unemployed
- *Healthcare professionals*- Practicing Field (spinal surgeon, specialist nurse, neuro-rehabilitation etc), years in practice, location, gender

Following confirmation of their eligibility to participate in the study, participants will be sent an on-line link to access the first round of the Delphi process. Instructions of how to complete the questionnaire will be included at the beginning of each round. Only participants who respond to the 1st round of the Delphi will be invited to participate in 2nd



For peer review only - http://bmjopen.bmj.com/site/about/guidelines.xhtml

round taking the assumption that if they had not participated in the first round they would be unwilling to participate in the second round. Data will be collected over at least a 4-week period for each round of the Delphi process. Participants who have not completed the survey will be sent reminders via email when they have 2 weeks, 1 week and 48 hours remaining for completion of the survey. Participants who have not completed the questionnaire within 4 weeks of the start will be deemed not to have completed that round of the Delphi. The language used by patients in the qualitative interviews will be used to help term the outcomes for the Delphi. Plain language summaries by the COMET Patient Participation, Involvement and Engagement (PoPPIE) group was used to develop the Delphi information sheet. The Delphi will be piloted with 2 participants from each stakeholder group to highlight any issues with understanding or validity.

**Scoring**

For an outcome to be included in the core outcome set there must be a majority agreement of the critical importance of the outcome and minority agreement that the outcome is not important [39]. This is in par with the GRADE (Grading of Recommendations Assessment, Development and Evaluation) working group recommendations (http://www.gradeworkinggroup.org; [40 41]. At the beginning of the Delphi, participants will be reminded the importance of completing the entire Delphi process. Round one of the Delphi study we will ask participants to rate each outcome using a 9 point Likert scale. This scoring system was chosen after previous studies and expert databases showed it differentiates the most between questionnaire items [16 37]. 7-9 indicates critical importance. 4 to 6 represents outcomes that are important but not critical whilst 1 to 3 are deemed to be of limited importance. All outcomes will be carried through to second round with anonymised feedback of first round scores from the patient group and from the healthcare professional group displayed for each outcome. The feedback will show the cumulated scores from each stakeholder group for each outcome and the participant will be asked to rate the outcomes again using the same 9 point Likert scale. If they change their score on the second round they will have the opportunity to explain their reasoning for this. Outcomes which have been suggested in round 1 by the participants and deemed appropriate by the study group will then be entered in for rating in the second round by key stakeholders. After the final Delphi round, there will be a list developed from all stakeholder



For peer review only - http://bmjopen.bmj.com/site/about/guidelines.xhtml

groups, which will be submitted to a face to face consensus meeting of key stakeholders to discuss what outcomes that should be finally included in the core outcome set. All participants who had completed both rounds of the Delphi survey will be eligible for invitation to the consensus meeting. A trained independent facilitator would chair this meeting.

**Analysis**

Consensus that an outcome should be included in the core outcome set is defined as 70% or more scoring it as 7 to 9 and fewer than 15% scoring it as 1 to 3, which is has been seen to be successful with the development of other core outcome sets [42 43] (**Table 4**). This will be done for each stakeholder group. Results at the multiple rounds of the Delphi process and consensus meeting will be documented to include the number of participants invited, number completing the section, measure of each group response to an outcome leading to a comprehensive list of all outcomes that should be included in the COS CES.

**Table 4**. Definitions of a consensus

| Classification of consensus | Description | Definition |
|---|---|---|
| **IN** | Consensus that outcome should be included in the core outcome set | 70% or more participants scoring as 7 to 9 AND <15% participants scoring as 1 to 3 in both stakeholder groups |
| **OUT** | Consensus that outcome should not be included in the core outcome set | 50% or less participants scoring 7 to 9 in both stakeholder groups |
| **NO CONSENSUS** | Uncertainty about importance of outcome | Anything else |

**Attrition**

It is expected that some participants will drop out after each round of the Delphi. Each participant will be given a unique participant number when they complete the first round of


For peer review only - http://bmjopen.bmj.com/site/about/guidelines.xhtml

the Delphi, which will allow calculation of the attrition rates between rounds. This will allow identification of participants who have completed all rounds and see if there is any difference bias between those participants who complete the process. Mean round 1 scores for the participants who completed round 1 and round 2 will be compared with those that dropped out after round 1.

***Phase 4: Consensus Meeting***

All participants registering for the Delphi survey will be asked if they would be happy to attend a face to face consensus meeting involving patients and healthcare professionals. They will need to complete both rounds of the Delphi survey to be eligible to attend. This would be set up as a tick box on the registration page for the online Delphi.
40 participants will be invited to the consensus meeting. This will include 20 healthcare professionals and 20 patients. Out of the 40 participants; 30 will be from the UK and 10 will be international. Standard travel expenses and hotel accommodation will be reimbursed or provided. 10 of the participants at the consensus meeting will be invited before the Delphi survey is released to attend the consensus meeting but on the premise, that both rounds of the Delphi are completed. This is to make sure there is representation at the consensus meeting from key stakeholder organisations closely involved with CES patients, research or management. 30 participants at the consensus meeting will be those who have completed both rounds of the Delphi and ticked their interest to attend the consensus meeting during registration.

In the development of a breast reconstruction core outcome set patients and professionals were recruited in a 2:1 ratio so that patients' views were represented preferentially as the procedure is a patient selected optional intervention [44]. In our study, clinical intervention for cauda equina syndrome is performed as an emergency so it was deemed appropriate by the study team to have a 1:1 ratio of patients and healthcare professionals. This is to maximise the number of participants involved to help achieve consensus. In addition, the core outcome set should reflect all key stakeholders input equally. If there is an overwhelming response with more than 40 participants interested in attending the consensus meeting the

For peer review only

study team will apply stratified purposive sampling. On the day of the consensus meeting informed consent will be obtained from the patient participants.

Outcomes categorised as “consensus in” across both stakeholder groups from the Delphi survey **(Table 4)** will be included in the final core outcome set. Outcomes categorised as “consensus out” across both stakeholder groups from the Delphi survey will be excluded from the final core outcome set. Results of the Delphi survey will be discussed at the consensus meeting and the main discussion will be regarding the outcomes deemed as achieving “no consensus” in the Delphi survey. Participants at the meeting will vote on these outcomes. The same criteria for consensus used in the Delphi survey **(Table 4)** will be used in the consensus meeting. All outcomes that reach “consensus in” will be included in the core outcome set. All outcomes in the “consensus out” or “no consensus” category after voting in the consensus meeting will not be included in the core outcome set. If there is no agreed final core outcome set at the end of the first meeting subsequent meetings will be arranged for this to happen. The participants who had completed both rounds of the Delphi survey would be invited to attend another consensus meeting if required.

**PATIENT INVOLVEMENT**

Patients will be involved in the design, review and recruitment of the study. The scope of the research question will be decided with the study team that includes 2 research partners who are patients with CES. The qualitative interviews will be trailed with the patient research partners and the topic guide will be reviewed by them. Pilot testing of the Delphi survey will be done by the patient research partners who will be asked to review the patient explanations of the outcomes and the questions on the registration page. Patients will be involved in the recruitment stage of the Delphi as they will be requested via social media to forward the website link for the Delphi survey to any relevant known contacts.

**ETHICS AND DISSEMINATION**

REC and HRA approval was obtained on the 6 December 2016 for the qualitative interviews from South Central - Hampshire A Research Ethics Committee. REC reference 16/SC/0587. REC and HRA approval was obtained on 26 March 2018 for the Delphi process and



For peer review only - http://bmjopen.bmj.com/site/about/guidelines.xhtml

consensus meeting from North West- Greater Manchester Central Research Ethics Committee. REC reference was 18/NW/0022. We intend to publish the results of the core outcome set for patients with CES in an open access journal. It will also be made available through the CES patient charity websites. Results will be disseminated through International and national presentations. The next step would be to identify the appropriate measurement instrument for each of the outcomes in the core outcome set [45]. Core outcome sets are developed in a number of clinical areas and their use is advocated in the UK by the National Institute for Health Research (NIHR) Health Technology Assessment (HTA), Cochrane Reviews of the effects of Healthcare intervention [46] and by World Health Organisation (WHO) handbook for guideline development [17]. The NIHR HTA has added this statement to their application form, "Where established core outcomes exist they should be included among the list of outcomes unless there is a good reason to do otherwise." By developing the CES core outcome set we intend to reduce outcome reporting bias, heterogeneity, and improve the quality of research studies in CES. This will allow us to synthesise the data and make more robust evidence based decisions regarding the management of CES.

**Author Contributions**

NS, MW, SC conceived the project. AGM is the principal investigator for the study. NS is the clinical research fellow responsible for management of the project, wrote the protocol and manuscript. AGM, PRW, AJN, MW, SC provide supervision, have input in all aspects of the project, commented on drafts of the manuscript. All authors have read and approved the manuscript.

**Acknowledgements**

Special thanks to Ms Claire Thornber and Mr Steven Smith as patient research partners in this study.

**Funding**

The corresponding author's research fellowship is funded by The Walton Centre Research Funds and The Royal College of Surgeons Research Fellowship.



For peer review only - http://bmjopen.bmj.com/site/about/guidelines.xhtml

**Competing Interests**

None Declared

**Supplementary file 1**- Topic guide

**Figure 1.** The overall study timeline

## REFERENCES

1. Kostuik JP. Controversies in cauda equina syndrome and lumbar disk herniation. *Current Opinion in Orthopaedics* 1993;4(2):125-28.
2. Gardner A, Gardner E, Morley T. Cauda equina syndrome: A review of the current clinical and medico-legal position. *Eur Spine J* 2011;20(5):690-97. doi: http://dx.doi.org/10.1007/s00586-010-1668-3
3. Gitelman A, Hishmeh S, Morelli BN, et al. Cauda equina syndrome: a comprehensive review. *Am J Orthop (Belle Mead NJ)* 2008;37(11):556-62.
4. Gleave JR, Macfarlane R. Cauda equina syndrome: what is the relationship between timing of surgery and outcome? *Br J Neurosurg* 2002;16(4):325-8.
5. Sorbie C. Cauda equina syndrome. *Orthopedics* 2009;32(6):397. doi: http://dx.doi.org/10.3928/01477447-20090511-31
6. Taskforce TNS. Commissioning Spinal Services - Getting The Service Back On Track . January 2013
7. Todd NV. Cauda equina syndrome: The timing of surgery probably does influence outcome. *Br J Neurosurg* 2005;19(4):301-06. doi: http://dx.doi.org/10.1080/02688690500305324
8. Srikandarajah N, Boissaud-Cooke MA, Clark S, et al. Does early surgical decompression in cauda equina syndrome improve bladder outcome? *Spine (03622436)* 2015;40(8):580-83. doi: 10.1097/BRS.0000000000000813
9. Ahn UM, Ahn NU, Buchowski JM, et al. Cauda equina syndrome secondary to lumbar disc herniation. *Spine* 2000;25(12):1515-22. doi: http://dx.doi.org/10.1097/00007632-200006150-00010
10. Daniels EW, Gordon Z, French K, et al. Review of medicolegal cases for cauda equina syndrome: what factors lead to an adverse outcome for the provider? *Orthopedics* 2012;35(3):200-00. doi: 10.3928/01477447-20120222-15
11. Williamson PR, Altman DG, Bagley H, et al. The COMET handbook: version 1.0. *Trials* 2017;18(3):280.
12. Armstrong R, Hall BJ, Doyle J, et al. 'Scoping the scope'of a cochrane review. *Journal of Public Health* 2011;33(1):147-50.
13. Williamson PR, Altman DG, Blazeby JM, et al. Developing core outcome sets for clinical trials: issues to consider. *Trials* 2012;13(1):132.
14. Kirkham JJ, Gargon E, Clarke M, et al. Can a core outcome set improve the quality of systematic reviews?–a survey of the Co-ordinating Editors of Cochrane Review Groups. *Trials* 2013;14(1):21.
15. Tugwell P, Boers M, Brooks P, et al. OMERACT: an international initiative to improve outcome measurement in rheumatology. *Trials* 2007;8(1):38.


For peer review only - http://bmjopen.bmj.com/site/about/guidelines.xhtml

16. COMET. The COMET Initiative. 2018
17. Williamson PR, Altman DG, Blazeby JM, et al. The COMET (core outcome measures in effectiveness trials) initiative. *Trials* 2011;12(Suppl 1):A70.
18. Kirkham JJ, Davis K, Altman DG, et al. Core Outcome Set-STAndards for Development: The COS-STAD recommendations. *PLoS medicine* 2017;14(11):e1002447.
19. Young B, Bagley H. Including patients in core outcome set development: issues to consider based on three workshops with around 100 international delegates. *Research Involvement and Engagement* 2016;2(1):25.
20. Srikandarajah N, Wilby M, Clark S, et al. Outcomes reported after surgery for Cauda Equina Syndrome: A Systematic Literature Review. *Spine* 2018
21. Moher D, Liberati A, Tetzlaff J, et al. Preferred reporting items for systematic reviews and meta-analyses: the PRISMA statement. *PLoS medicine* 2009;6(7):e1000097.
22. Kostuik JP. Medicolegal consequences of cauda equina syndrome: an overview. *Neurosurg* 2004;16(6):e8.
23. Dodd S, Clarke M, Becker L, et al. A taxonomy has been developed for outcomes in medical research to help improve knowledge discovery. *Journal of clinical epidemiology* 2018;96:84-92.
24. Keeley T, Khan H, Pinfold V, et al. Core outcome sets for use in effectiveness trials involving people with bipolar and schizophrenia in a community-based setting (PARTNERS2): study protocol for the development of two core outcome sets. *Trials* 2015;16(1):47.
25. Elo S, Kyngäs H. The qualitative content analysis process. *Journal of advanced nursing* 2008;62(1):107-15.
26. Quinn Patton M. Qualitative Research John Wiley & Sons: Ltd, 2005.
27. Gleave JRW, Macfarlane R. Cauda equina syndrome: What is the relationship between timing of surgery and outcome? *Br J Neurosurg* 2002;16(4):325-28. doi: http://dx.doi.org/10.1080/0268869021000032887
28. Ritchie J, Lewis J, Nicholls CM, et al. Qualitative research practice: A guide for social science students and researchers: Sage 2013.
29. Britten N. Qualitative research: qualitative interviews in medical research. *Bmj* 1995;311(6999):251-53.
30. Braun V, Clarke V. Using thematic analysis in psychology. *Qualitative research in psychology* 2006;3(2):77-101.
31. Charmaz K. Constructing grounded theory: A practical guide through qualitative analysis: Sage 2006.
32. Smith JA, Flowers P, Larkin M. Interpretative phenomenological analysis: Theory, method and research2009.
33. Tong A, Sainsbury P, Craig J. Consolidated criteria for reporting qualitative research (COREQ): a 32-item checklist for interviews and focus groups. *International journal for quality in health care* 2007;19(6):349-57.
34. Khurana R, Arora SK, Hemal A, et al. Successful non-operative management of cauda equina syndrome in a case of thalassemia major. *Pediatric Hematology Oncology Journal* 2016;1(2):41-43.
35. Keeley T, Williamson P, Callery P, et al. The use of qualitative methods to inform Delphi surveys in core outcome set development. *Trials* 2016;17(1):230.
36. Hasson F, Keeney S, McKenna H. Research guidelines for the Delphi survey technique. *Journal of advanced nursing* 2000;32(4):1008-15.
37. Sinha IP, Smyth RL, Williamson PR. Using the Delphi technique to determine which outcomes to measure in clinical trials: recommendations for the future based on a systematic review of existing studies. *PLoS medicine* 2011;8(1):e1000393.



For peer review only - http://bmjopen.bmj.com/site/about/guidelines.xhtml

38. Murphy M. Consensus development methods and their use in clinical guideline development. *Health Technol Assess* 1998;2:1-88.
39. Jaeschke R, Guyatt GH, Dellinger P, et al. Use of GRADE grid to reach decisions on clinical practice guidelines when consensus is elusive. *BMJ: British Medical Journal (Online)* 2008;337
40. Atkins D, Best D, Briss PA, et al. Grading quality of evidence and strength of recommendations. *BMJ (Clinical research ed)* 2004;328(7454):1490-90.
41. Guyatt GH, Oxman AD, Vist GE, et al. GRADE: an emerging consensus on rating quality of evidence and strength of recommendations. *BMJ (Clinical research ed)* 2008;336(7650):924-26.
42. Harman NL, Bruce IA, Callery P, et al. MOMENT–Management of Otitis Media with Effusion in Cleft Palate: protocol for a systematic review of the literature and identification of a core outcome set using a Delphi survey. *Trials* 2013;14(1):70.
43. Haywood K, Griffin X, Achten J, et al. Developing a core outcome set for hip fracture trials. *Bone Joint J* 2014;96(8):1016-23.
44. Potter S, Holcombe C, Ward J, et al. Development of a core outcome set for research and audit studies in reconstructive breast surgery. *British Journal of Surgery* 2015;102(11):1360-71.
45. Prinsen CA, Vohra S, Rose MR, et al. How to select outcome measurement instruments for outcomes included in a "Core Outcome Set"–a practical guideline. *Trials* 2016;17(1):449.
46. Rosenbaum SE, Glenton C, Oxman AD. Summary-of-findings tables in Cochrane reviews improved understanding and rapid retrieval of key information. *Journal of clinical epidemiology* 2010;63(6):620-26.

For peer review only

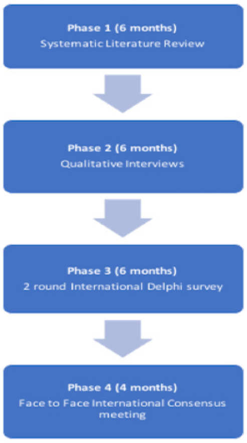


The overall study timeline in four phases with the estimated timeframes.

90x90mm (300 x 300 DPI)

IRAS: 201946 Version: 1.1 Date: 23/09/16

# TOPIC GUIDE CES QUALITATIVE INTERVIEWS

## Aims and Objectives

-To explore the patient experience of living with Cauda Equina Syndrome (CES)
-To ascertain what the patient feels are the most important outcomes that they are experiencing
-To ascertain what outcomes the patient feels are the most important to research in to improve CES management and aftercare
-To determine who should be key stakeholders
-Identify appropriate language to use for patient Delphi iterative process.

## Introduction (5-10 mins)

Interviewer Name
Interviewer Occupation
Explain basic definition of CES
Explain looking for challenges experienced after the operation for CES
Explain expected intention, sensitive subjects and duration of interview and confidentiality
Confirm consent to qualitative interview

## Background (<5 mins)

Interviewee name
Interviewee age
Interviewee occupation
Other medical conditions
When was your operation for CES?

## Interview questions (30 mins)

How has your experience of this condition; Cauda Equina Syndrome been?
- What was it like before the back operation?
- What was it like after the back operation?

How do you feel your condition has been managed in hospital and in the community?

What were your expectations of life health-wise after the operation and what is the reality like?

For peer review only

IRAS: 201946 Version: 1.1 Date: 23/09/16

Due to this condition what do you feel are the challenges to your health and wellbeing?
-bowel/bladder
-sex life
-back/ leg pain
-psychological
-anxiety/fear
-other

Would you be able to prioritise the importance of these for you now?

Was the importance of these different at earlier stages of the condition? (More relevant to those in the long term CES category)

Through this process of living with CES who else do you think has a good handle on the condition? If anyone? -Gauge other potential key stakeholders

Tell me a bit about the support you had for the condition?

**Closing remarks (5 mins)**

Considering your hospital, post op and follow up experience what would you have liked to change?
-support services
-more streamlined service with dedicated clinics
-research into timing for CES operations
-follow up as to the effects of long term CES

Offer the opportunity for the participant to comment on their interview transcript after transcription.

For peer review only
